# Comparative Analysis of HEK293 Genomic Variability

**DOI:** 10.1002/bit.70105

**Published:** 2025-11-10

**Authors:** Georg Smesnik, Nikolaus Virgolini, Maria Toth, Astrid Dürauer, Nicole Borth

**Affiliations:** ^1^ Institute of Bioprocess Science and Engineering, Department of Biotechnology and Food Science CD Laboratory of Knowledge‐Based Production of Gene Therapy Vectors, BOKU University Vienna Austria; ^2^ Institute of Bioprocess Science and Engineering, Department of Biotechnology and Food Science BOKU University Vienna Austria; ^3^ Institute of Animal Cell Technology and Systems Biology, Department of Biotechnology and Food Science BOKU University Vienna Austria

**Keywords:** adaptation to suspension, DNA variant analysis, genomics, HEK293, whole‐genome sequencing

## Abstract

Human embryonic kidney cells HEK293 are widely used in biopharmaceutical manufacturing, with a recent surge particularly in recombinant adeno‐associated virus production. Despite their industrial relevance, comprehensive data on their genomic background and stability remains limited. Here, we systematically analyze the genetic landscape of various HEK293 cell lines in response to cultivation conditions, clonal selection, genetic manipulation and over time in culture. Adherent HEK293 were adapted to suspension growth using different serum‐free media. Whole genome sequences from these cell lines were analyzed together with previously published data from additional variants in common use. All data sets were aligned against the human reference genome, enabling the assessment of genome stability by evaluation of variants and revealing a conserved genetic core across all lines, regardless of cultivation history or phenotypic divergence. Evaluation of the functional implications of conserved core mutations identified an enrichment in genes related to cellular structure, morphology and cellular connectivity. The distribution of structural variants and single nucleotide polymorphisms indicated a gradual accumulation of mutations over time in culture rather than abrupt shifts in response to environmental changes. Notably, the integrated adenoviral genes remained highly conserved with respect to copy number, integration site and sequence integrity. These findings provide insight into the genomic evolution of HEK293 cells and offer a foundation for further multi‐omics studies aimed at optimizing HEK293 cells for applications in biopharmaceutical production.

## Graphical abstract

An investigation of how HEK293‐derived cell lines adapt genetically to different culture conditions and environmental pressures: despite distinct phenotypes and cultivation histories, comparative whole‐genome analyses of established HEK293 variants as well as newly suspension‐adapted cells revealed a shared, common set of mutations linked to cell structure and connectivity. The findings show that genomic alterations accumulate gradually over time, indicating continuous drift rather than abrupt shifts in response to environmental changes.

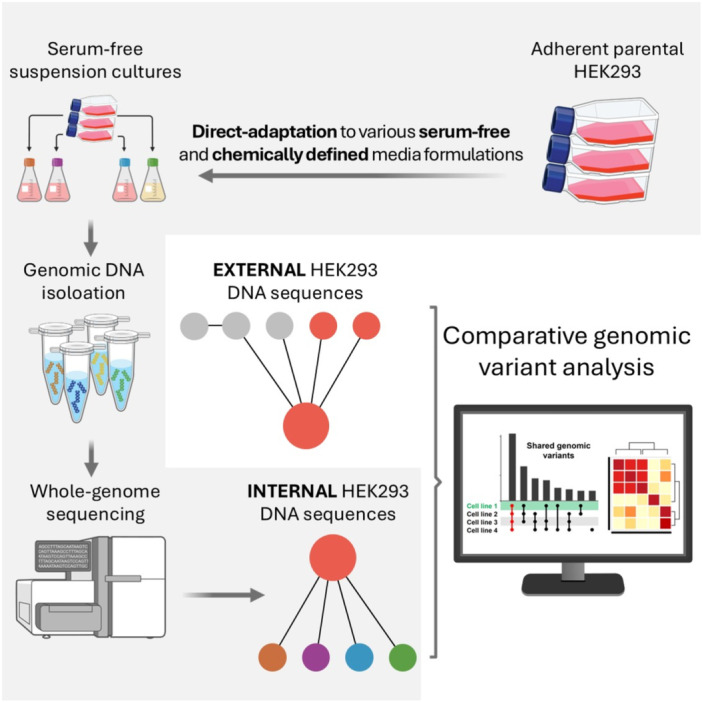

## 1 Introduction

The human embryonic kidney cell line (HEK293) has been widely used for over 50 years in research applications ranging from signal‐transduction and protein‐interaction studies to small‐scale protein expression systems. More recently, it has seen renewed interest as a large‐scale biopharmaceutical production platform for viral products. HEK293 cells originate from the kidney of an aborted human female embryo and were first immortalized in 1973 through the integration of a 4 kb fragment of the human adenovirus 5 genome (HAdV.5) into chromosome 19 (Russell et al. 1977; Louis et al. 1997; Graham and van der Eb 1973). The expression of adenoviral genes early region 1 A (E1A) and early region 1B (E1B) inhibits apoptosis and modulates transcription and cell cycle regulation, permitting continuous culturing of the cell line. As is characteristic for immortalized cell lines, HEK293 cells exhibit a continuously evolving genome with chromosomal translocations and copy number alterations, resulting in a pseudotriploid karyotype (Stepanenko and Dmitrenko 2015; Bylund et al. 2004).

On top of the genetic variation expected of an immortalized cell line, different lineages with specific pre‐history exist of HEK293. These include adherent cultures, such as HEK293T and HEK293E, along with the original parental HEK293 cell line from the American Type Culture Collection (ATCC CRL‐1573). HEK293T and HEK293E were engineered for improved transgene expression by introducing viral elements. The HEK293T lineage expresses a temperature‐sensitive allele of the Simian Virus 40 (SV40) large T antigen, while the HEK293E lineage carries the Epstein‐Barr virus nuclear antigen EBNA1 (DuBridge et al. 1987; Swirski et al. 1992). In addition to the adherent lineages, several suspension‐adapted derivatives, both as pools and subclones are available commercially or internally at different research labs. These include HEK293_6E, which carries a truncated EBNA1 expression system, as well as the commercially available HEK293F and HEK293H (Gibco, Thermo Fisher Scientific), both optimized for enhanced suspension growth. Additionally, HEK293 Freestyle was derived from HEK293F through adaptation to Freestyle medium (Gibco, Thermo Fisher Scientific). These sub‐lineages were developed to enhance recombinant protein production and support manufacturing of therapeutic products (Lalonde and Durocher 2017; Dumont et al. 2016). As renewed interest in viral‐based gene therapies grows, HEK293 has regained attention as a promising production platform, due to its integrated adenoviral genes that serve as essential helper factors for the synthesis of adeno‐associated virus vectors (rAAV). The prominence of rAAV‐based products as advanced clinical applications highlights the potential of HEK293 cells in biopharmaceutical manufacturing. Although this platform has demonstrated suitability for commercial‐scale production, its efficiency and scalability remain constrained by intrinsic cellular limitations and variability.

In contrast, extensive and systematic genome scale host cell characterization of Chinese hamster ovary cells (CHO) as the mammalian platform for recombinant protein production has facilitated remarkable progress in identifying and overcoming limiting factors within fundamental biological processes (Sellick et al. 2011; Stolfa et al. 2018; Kildegaard et al. 2013). However, while comprehensive omics profiles have become available for CHO cells over the last 15 years, genome‐wide studies characterizing HEK293 cells have been limited to date (Lin et al. 2014; Malm et al. 2020). As such, a deeper understanding of the fluidity of these molecular processes, not only over culture time, but also due to evolutionary and environmental conditions is essential, to guarantee the stability of HEK293‐based productions and to facilitate targeted optimization both of process and cell line.

For the purpose of this study, several sub‐lineages were generated in‐house by adaptation of an adherent HEK293 ATCC cell line to suspension growth in different serum‐free, chemically defined media formulations and subsequently whole‐genome sequenced, along with the parental adherent cell line and a commercially available control (HEK293_6E). In addition to these newly generated data, we used recently published genome sequences of six clonally derived HEK293 cell lines from a previous study by Malm et al (Malm et al. 2020). In total, genome sequences of 13 samples of HEK293 derived cell lines, each established through distinct manipulations and/or selection conditions, are included in our comparative study. The examination of genomic variations across these HEK293 cell lines was performed by a standardized and reproducible data analysis workflow designed for comprehensive variant detection in whole‐genome datasets. Variant calling was carried out to identify both small variants such as single‐nucleotide polymorphisms (SNP) and small insertions and deletions (indel) as well as for larger structural rearrangements (SV) such as translocations, duplications or large insertions and deletions. These genomic variations were systematically compared across all samples to identify shared and unique mutations and assess their potential correlation with phenotypic traits and functional characteristics. Moreover, a genome‐wide analysis of karyotypic changes and copy number alterations was conducted to assess genomic stability across all samples. Specifically, we investigated whether the integration sites and copy numbers of human adenovirus 5 (HAdV.5) sequences were conserved across samples, as variations in these regions may specifically influence performance and productivity.

## 2 Results

### 2.1 Direct Adaptation of Adherent HEK293 Cell Line to Serum‐Free Suspension Conditions

**Figure 1:**
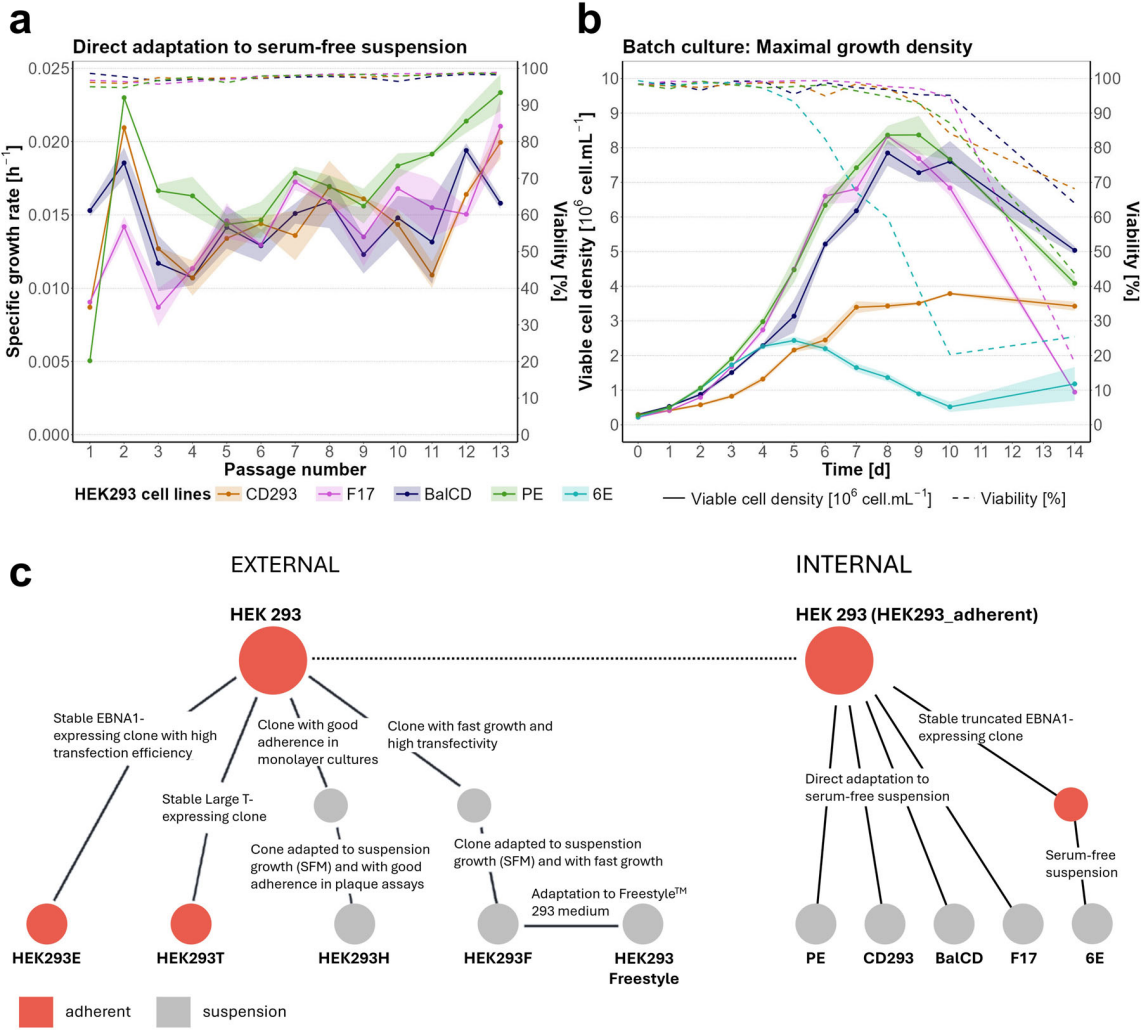
HEK293 cell line adaptation, growth performance and sample relationships. (a) Specific growth rates and viability profiles over the course of adaptation from adherent cultivation to serum‐free suspension conditions in four different media formulations: Gibco CD293 medium (CD293), Gibco Freestyle F17 medium (F17), Fujifilm BalanCD HEK293 medium (BalCD), Cytiva HyClone Peak Expression medium (PE). (b) Cell growth and viability profiles of adapted cell lines and HEK293_6E cell line (6E), serving as a suspension reference, during a 14‐day batch cultivation. Shading represents the standard deviation of 3 biological replicates. (c) Schematic overview of relationship and developmental stage of publicly available sequencing data (left) and internal cell lines (right) used in this comparative genomic analysis. Red dots indicate adherent cultivation conditions, gray dots mark suspension conditions. Figure was modified from the original study (Malm et al. 2020).

To generate serum‐free suspension‐adapted cell lines from the original adherent HEK293 cells, a direct adaptation approach was applied, consisting of removal of serum and transition into suspension cell growth in a single step. The process was performed in small‐scale shaking tubes using the respective chemically defined media and by maintaining independent parallel cultures for each of the four conditions over an 8‐week period. Throughout this time, the cultures were subcultured twice per week, and specific growth rates and viability were continuously monitored (Figure 1a). Despite the abrupt removal of serum and adaptation to suspension growth, cell viability remained high, never dropping below 95% in any culture. In addition, all four adapted cultures exhibited comparable growth rates with only minor variations. Throughout the adaptation process, specific growth rates gradually increased, ultimately reaching 0.020 h⁻¹, which is slightly lower than the typical range of 0.025 to 0.030 h⁻¹ observed in adherent HEK293 cells (data not shown). The resulting suspension‐adapted cell lines were designated according to the respective media used: Cytiva HyClone peak expression medium (HEK293_PE), Gibco CD293 medium (HEK293_CD293), Fujifilm BalanCD HEK293 medium (HEK293_BalCD), Gibco FreeStyle F17 medium (HEK293_F17). Importantly, to differentiate between the respective contribution of adventitious changes in genome sequence and the evolutionary pressure exerted by the respective medium, cells were adapted to the HyClone peak expression medium twice, in two independent, parallel cultures, labeled HEK293_PE_p1 and HEK293_PE_p2.

To compare the growth phenotype of our established cell lines to the suspension cell line HEK293_6E, a 14‐day batch culture experiment was conducted (Figure 1b). The results revealed substantial differences in growth performance across cell lines. HEK293_PE, F17 and BalCD achieved significantly higher cell densities (8.6 ± 0.6 × 10^6^ cell. mL^−1^, 8.3 ± 0.1 × 10^6^ cell. mL^−1^, and 7.9 ± 0.7 × 10^6^ cell. mL^−1^ respectively) compared to HEK293_CD293 and HEK293_6E, reaching their maximum viable cell densities between day 8 and 10. In contrast, HEK293_6E indicated a decreased growth capacity, peaking at 2.4 ± 0.2 × 10^6^ cell. mL^−1^ on day 5 of the batch cultivation. Thus, the established cell lines, with the exception of HEK293_CD293 revealed an improved growth phenotype compared to HEK293_6E.

### 2.2 Variant Calling for All Available Genome Sequences

Genomic DNA was isolated from adherent ATCC HEK293 cells, HEK293_6E cells and from all four suspension adapted cell lines including the two adaptations into PE. All DNA samples were sequenced and the resulting data sets processed together with data generated in a previous study by Malm et al. (NCBI, PRJNA565658) (Malm et al. 2020). Both sets include cell lines cultivated under adherent conditions, namely, HEK293, HEK293_adherent, HEK293E, and HEK293T, as well as suspension conditions, including HEK293H, HEK293F, HEK293Freestyle, HEK293_6E, HEK293_PE, HEK293_CD293, HEK293_BalCD, and HEK293_F17 (Figure 1c). Thus, in total thirteen samples of twelve HEK293 cell lines with different grades of relationship or previous history were analyzed together, to gain a deeper understanding of HEK293's genomic landscape and its plasticity throughout evolutionary pressures. Furthermore, including sequencing data from two distinct research groups, containing the same reference cell line (e.g. ATCC HEK293 adherent) also serves to assess potential genomic differences in cell lines cultivated and/or generated under similar conditions in different locations.

**Figure 2:**
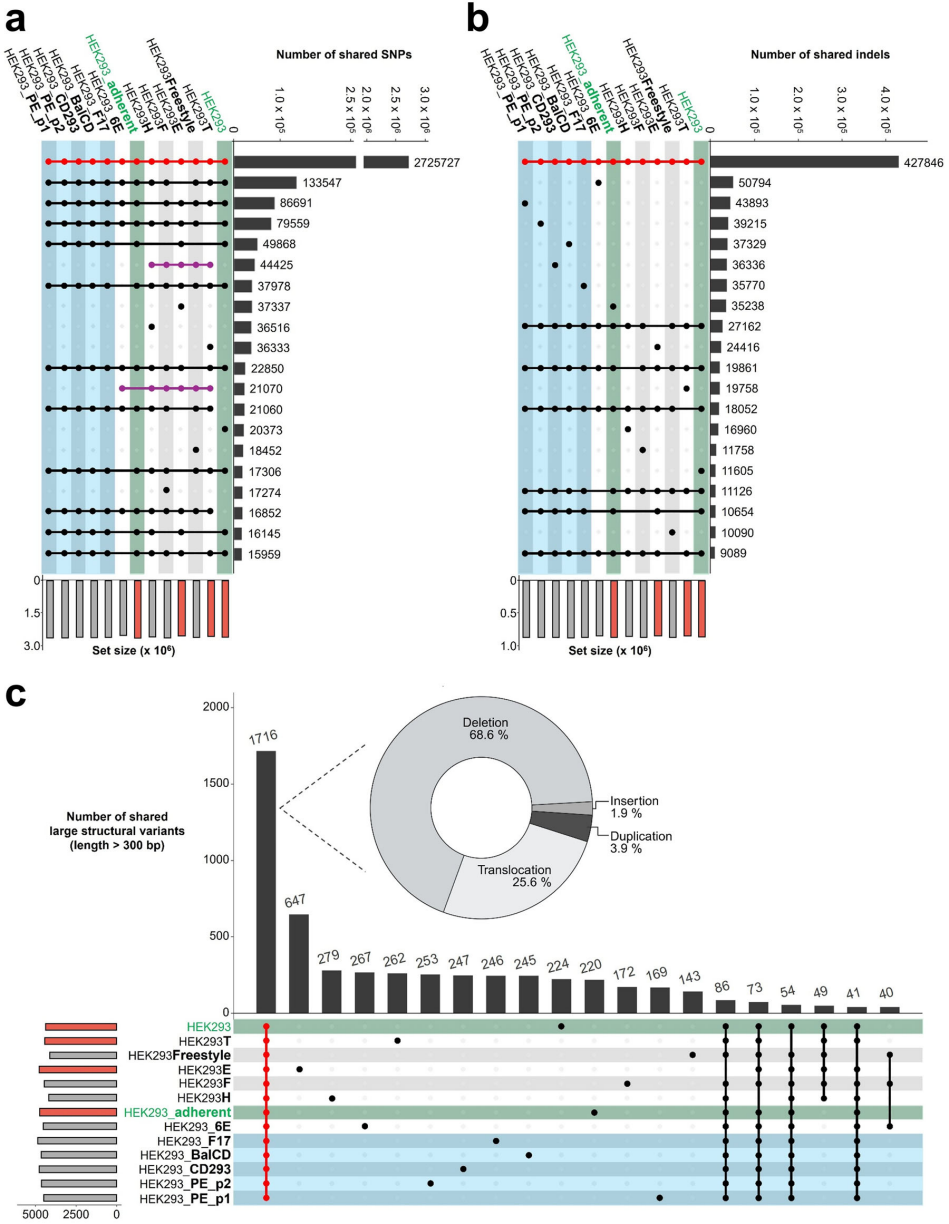
Comparative analysis of small‐ and structural variants. Upset plots reveal the top 20 combinations of shared SNPs (a), indels (b) and large structural variants (c) across all evaluated samples. Number of variants per sample (set size) is indicated by light gray bars (suspension cell lines) and light red bars (adherent cell lines) below (a, b) or next to the matrix (c). Dots and connecting lines in the matrix show the variants shared between samples, with their number shown by the corresponding black bar. Matrix shading corresponds to sample type: green shading marks the two ATCC derived parental cell lines, blue shading marks in‐house suspension‐adapted cell lines. Variant combination shared by all samples are highlighted in red, those exclusively shared by genetically modified or clonally derived cell lines are highlighted in purple. (c) Donut chart shows variant type distribution among large structural variants shared by all samples.

A systematic workflow was applied to the sequencing data of all cell lines to comprehensively characterize genetic variations. These were analyzed for shared and unique mutations, as well as for potential associations with phenotypical conditions. On average, roughly 3.5 million SNPs were identified per sample, of which over 2.7 million (about 77%) were shared across all genomes (Figure 2a). This indicates a characteristic genetic signature common to all HEK293 cells, independent of their cultivation conditions or phenotypic divergence. Similarly, the analysis of indels shows a substantial core set of over 400,000 indels present in all samples (Figure 2b), supporting the assumption of a conserved genomic background in HEK293 cells. Although the observed clustering patterns do not allow a direct linkage to specific cultivation conditions, closely related derivatives, such as suspension‐adapted cell lines, exhibit a higher degree of shared variants for both SNPs and indels (Figure 2a,b). In contrast, cell lines which had undergone genetic modifications such as HEK293T and HEK293E or the clonally derived cell line HEK293H have more unique variants, reflecting these targeted genetic alterations and longer historical divergence. Moreover, the presence of unique SNP and indel subsets distributed across different sub‐lineages, and to a lesser extent between the two parental adherent cell lines, indicate a gradual accumulation of genetic changes over time in culture. This pattern may suggest a continuous rate of mutation rather than abrupt genomic shifts in response to altered cultivation conditions. This interpretation is further supported by the variant appearance rates observed over the 8‐week direct‐adaptation process (Supporting Information Figure S1). During this period, each cell line acquired mutations at a similar rate relative to the parental HEK293_adherent. Comparable numbers of unique, new variants in the parallel adaptations HEK293_PE_p1 and HEK293_PE_p2 also indicate a consistent rate of randomly arising mutations over time.

To further extend the genomic analysis, larger structural rearrangements were evaluated. As such, a stringent size filtration criterion of at least 300 base pairs was applied, allowing to prioritize the identification of larger, more reliable structural changes while minimizing the risk of false positives. Additionally, it highlights variants that may be more likely to have biological significance or impact genomic integrity. Compared to small variants, larger structural variants (SV) occur less frequently (appearance rates of approximately one per 700,000 base pairs, compared to 1 in 3000 to 4000 bp for indels and 1 in 900–1000 bp for SNPs), however they exhibit greater variability among cell lines (Supporting Information Tables S1, S2 and Figure S2). Comparison of SVs again reveals a substantial fraction consistently present across all samples, further substantiating the HEK293 specific genetic background. Of these, deletions are the most abundant type, followed by translocations, while duplications and insertions are rarely detected (Figure 2c). Despite the presence of a large set of 1716 shared SVs, minimal overlap is observed within specific subgroups (e.g. parental adherent or in‐house suspension‐adapted). However, individual cell lines display unique SV sets of comparable sizes. Notably, HEK293E exhibits a distinctive SV profile, with an increased number of translocations and insertions compared to other cell lines (Figure 2c). This pattern aligns with the previously observed trend in SNPs and indels, where engineered cell lines share fewer variants with other HEK293 derivatives.

### 2.3 Evaluation of Potential Biological Impact of Genome Variants

To assess the potential biological impact of identified genomic variants, gene ontology (GO) enrichment was assessed to selected subsets of variants to estimate their impact on biological processes or cellular components. Three subsets of variants were investigated: variants of all types (SNP, indel, SV) shared by all 13 samples, hereafter referred to as common core mutations; variants of all types absent in both parental adherent cell lines (HEK293, HEK293_adherent) but present in one or more suspension cell lines, referred to as suspension‐associated variants; and size‐filtered structural variants shared by all 13 samples, referred to as common large structural rearrangements.

For the common core mutations and suspension‐associated variants, only those predicted to have high or moderate impact according to the variant annotation were included in the enrichment analysis. High‐impact variants are most likely disruptive (e.g., stop‐gain, frameshift, splice donor/acceptor changes), while moderate‐impact variants include missense mutations (amino acid substitutions) or in‐frame indels that might affect protein function (Cingolani et al. 2012; McLaren et al. 2016) For all three variant categories, both homozygous and heterozygous mutations were included in the GO enrichment analysis to comprehensively capture potential functional effects.

Analysis of common core mutations showed significant enrichment in genes involved in biological processes related to cell structure and cytoskeletal organization (GO:0045104, GO:0045109, GO:0005938, GO:0030864, GO:0098858, GO:0016459), cell adhesion and extracellular matrix components (GO:0098742, GO:0016339, GO:0030198, GO:0005604, GO:0030199), as well as sensory functions (GO:0007608). The most enriched cellular component was the extracellular matrix (GO:0062023), a structure essential for mechanical stability and the transmission of biochemical signals (Supporting Information Figure S2, Supporting Information Table S3) (Frantz et al. 2010).

Further analysis of the 1716 common large structural rearrangements revealed that over 70% were located in gene‐ or transcript‐associated regions, while approximately 22% were found in intergenic or intronic regions (Figure 2c). These variants, representing a specific subset of common core mutations, showed enrichment for genes involved in nervous system development and neural connectivity. The most prominently enriched biological processes included synapse organization (GO:0050807), neuron projection guidance (GO:0097485), and developmental growth involved in morphogenesis (GO:0060560). Enriched cellular components were predominantly related to synaptic and postsynaptic membrane molecules (GO:0097060, GO:0045211). Overall, structural mutations in the associated genes are linked to cellular organization, connectivity, and morphogenetic functions, commonly attributed to neuronal and epithelial‐like features (Supporting Information Figure S3, Supporting Information Table S4) (Gene Ontology 2023; Binns et al. 2009).

Although several genes associated with cell adhesion processes were also found enriched in both the common core mutations and common large structural rearrangements categories, this enrichment was particularly pronounced in the suspension‐associated category. Enrichment analysis revealed a single enriched biological process: homophilic cell adhesion via plasma membrane adhesion molecules (GO:0007156) with 45 significantly enriched genes identified (Supporting Information Tables S5 and S6). All 45 genes were found to belong either to the immunoglobulin family, such as cell adhesion molecules and contactins, or to the cadherin superfamily, including desmocollins, desmogleins, and protocadherins, consistent with previous observations based on external data alone (Malm et al. 2020). A moderate reduction of significance thresholds (method: Benjamini and Hochberg, *p*‐value cutoff: 0.08, q‐value cutoff: 0.30) revealed enrichment of additional terms related to cellular connectivity (GO:0007416, GO:0098742), cytoskeletal organization and cell shape regulation (GO:0031252, GO:0005938) (Supporting Information Figure S4, Supporting Information Table S7).

### 2.4 Copy Number Aberrations

**Figure 3:**
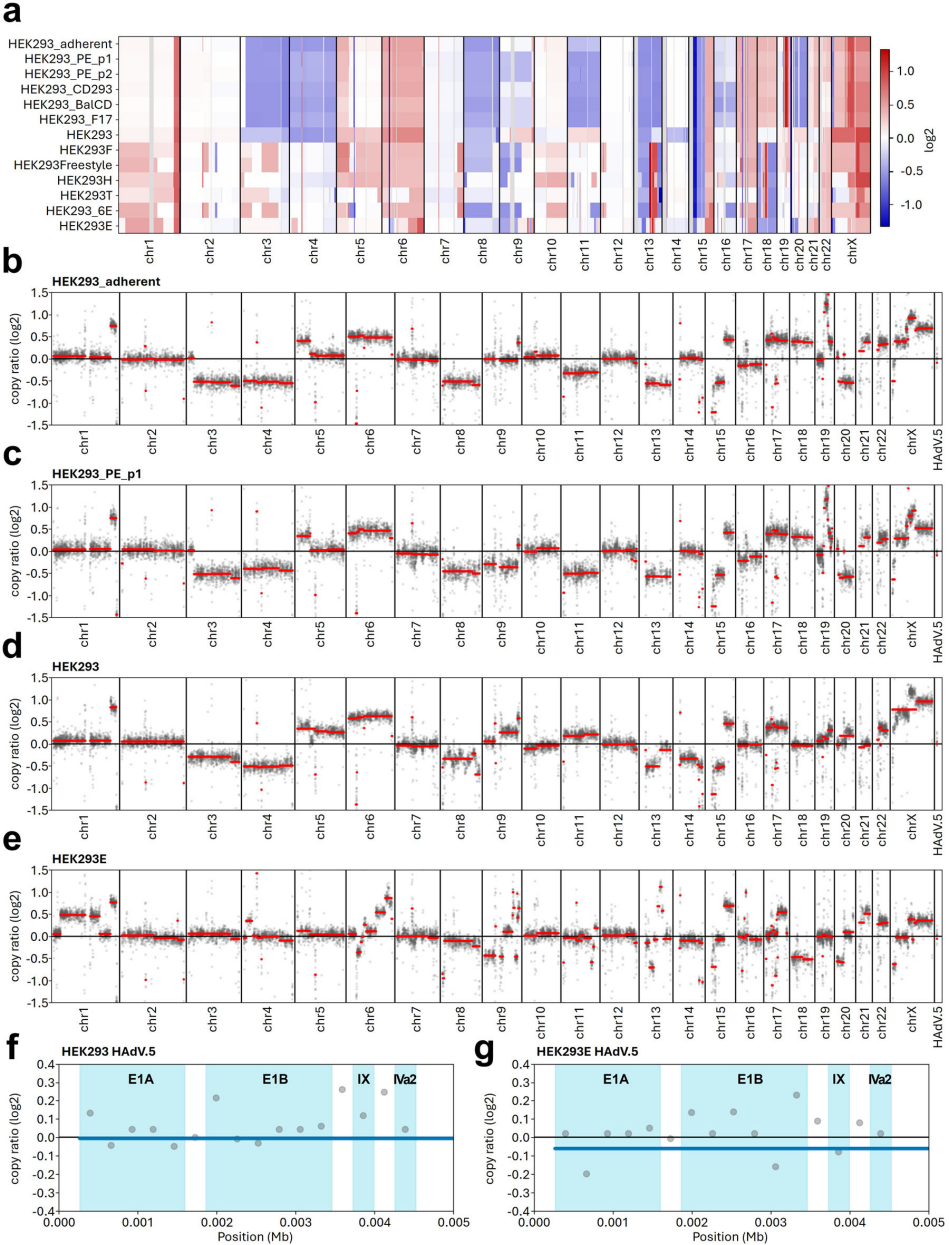
Genome‐wide and HAdV.5‐specific copy number variation profiles. Heatmap of genome‐wide copy number gains (red) and losses (blue) across all cell lines relative to a diploid reference (a) Individual genome‐wide CNV profiles, relative to a diploid reference, exemplarily shown for parental HEK293 cell lines (b, d), the direct‐suspension‐adapted HEK293_PE_p1 (c) and the genetically modified HEK293E cell lines (e). CNV profiles are shown as scatter plots of calculated coverage bins across the observed genomic region, with panel width proportional to its sequence length. Genome‐wide panels (b–e) display the entire human genome (chr1‐chr22, chrX) and the additional human adenovirus 5 scaffold (HAdV.5). HAdV.5‐specific panels of HEK293 (f) and HEK293E (g) reveal the integrated 4 kb adenoviral segment with light blue areas indicating the open reading frames of corresponding viral genes: early region 1A (E1A), early region 1B (E1B), protein IX (IX) and intermediate‐early transcript IVa2 (IVa2). Values are presented on a log2 scale, with 0 corresponding to the diploid state of the reference, positive ratio indicate gains, and negative ratio indicate losses in the sample. Trend in copy number alteration is highlighted by a red (b–e) or blue (f, g) horizontal line. Analysis was performed using the flat‐reference option (detailed described in Supporting Information S1).

To assess how identified large structural rearrangement events are potentially impacted by known aberrations in HEK293 chromosome numbers, copy number alterations across the genome were analyzed, assuming a normal, diploid karyotype as reference (Bylund et al. 2004; Lin et al. 2014; Stavropoulou et al. 2005; Stepanenko et al. 2015). Numerous large‐scale deletions and duplications were revealed (Figure 3b–e and Supporting Information Figures S5 to S11), consistent with the reported pseudo‐triploid karyotype of HEK293 (Stepanenko and Dmitrenko 2015). Despite these apparent chromosomal imbalances, similar copy number variation (CNV) profiles were detected for closely related cell lines, such as suspension adapted cultures and their adherent parent. In contrast, cell lines that went through extensive manipulation, for instance by additional viral transformations and selection mechanisms (e.g. HEK293E, HEK293_6E and HEK293T), reveal distinct CNV profiles, sharing fewer similarities with all other evaluated cell lines (Figure 3a). This is exemplarily demonstrated in Figure 3b–e, where adherent HEK293 cell lines (Figure 3b,d) are compared to either a serum‐free suspension adapted cell line HEK293_PE_p1 (Figure 3c) or the EBNA1‐expressing adherent cell line HEK293E (Figure 3e). Although the resolution of this global analysis is limited to larger alterations, the comparison highlights notable genome‐wide differences. As illustrated in Figure 3b to e and further described below, the suspension‐adapted cell line HEK293_PE_p1, directly derived from HEK293_adherent, shows greater similarity to its parent as well as to the other parental adherent line, HEK293. In contrast, HEK293E exhibits prominent alterations compared to both original HEK293 cell lines (HEK293 and HEK293_adherent). In more detail, while a diploid copy number pattern with a large, amplified region at the end of chromosome 1 is observed in parental adherent‐ and suspension‐adapted samples, genetically modified (HEK293_6E, HEK293E, HEK293T) or clonally derived (HEK293H, HEK293F) cell lines show an additional extensive amplification across large portions of this chromosome (Figure 3b–e and Supporting Information Figures S5 to S11). Moreover, chromosomes 3 and 4 display highly similar CNV profiles among closely related parental adherent (HEK293_adherent) and directly derived suspension adapted (HEK293_PE_p1, HEK293_PE_p2, HEK293_CD293, HEK293_BalCD and HEK293_F17) cell lines (Figure 3b–c and Supporting Information Figures S5 to S8), characterized by a chromosome‐wide decrease in gene copy number, whereas genetically modified or clonally derived samples exhibit a more diploid‐like pattern in these regions (Figure 3e and Supporting Information Figures S8 to S10). Of particular interest are the patterns observed for chromosomes 6 and 19. For chromosome 6, which has been shown to be the most variable chromosome in copy number in karyotype studies (Bylund et al. 2004; Binz et al. 2019), parental adherent and derivative suspension‐adapted samples exhibit a moderate increase in copy number (Figure 3b,c), whereas HEK293E shows a widespread variability ranging from a onefold decrease to a twofold increase across the chromosome (Figure 3e). Conversely, for chromosome 19, the reported integration site of adenoviral genes, the adherent and derivative suspension‐adapted in‐house samples display various amplifications, while HEK293E maintains a more consistent, near diploid profile, marked by numerous smaller alterations in both directions (Figure 3b–e) (Louis et al. 1997).

### 2.5 Integration Pattern, Copy Number Variation and Sequence Integrity of Adenoviral Genes

Given the observed high variability of CNV profiles on chromosome 19, it was of particular interest to assess the CNV patterns of the integrated adenoviral genes, due to their reported essential role in maintaining the immortalized state of the cell line (Moran and Mathews 1987; Flint and Shenk 1997; Cuconati and White 2002; Reich et al. 1988; Bhattacharya et al. 1996; Zhao et al. 2003). Therefore, we next focused on the human adenoviral scaffold region, specifically on reads that are fully aligned to the viral sequences of the combined reference. Analysis of viral sequences revealed coverage of the expected adenoviral regions, spanning over the first 4344 base pairs on the human adenovirus 5 genome (HAdV.5) (Russell et al. 1977; Louis et al. 1997). These include the early viral genes early region 1 A (E1A) and early region 1B (E1B), the delayed early genes protein IX (IX) and the intermediate‐early transcript IVa2 (IVa2), as well as truncated fragments of the inverted terminal repeat (ITR) sequences located at both ends of the viral genome (Chroboczek et al. 1992; Davison et al. 2003). The average predicted copy number in these regions remains close to the diploid baseline (log2 ratio of 0.0), with bins, each representing the calculated copy number for a 267 bp stretch of DNA ranging from ‐0.2 to 0.3 on the log2 scale (Figure 3f,g). A comparison of CNV profiles across all analyzed samples confirms a stable copy number of these genes, suggesting a highly conserved structure at the adenoviral integration site (Supporting Figures 12 to 15).

**Figure 4:**
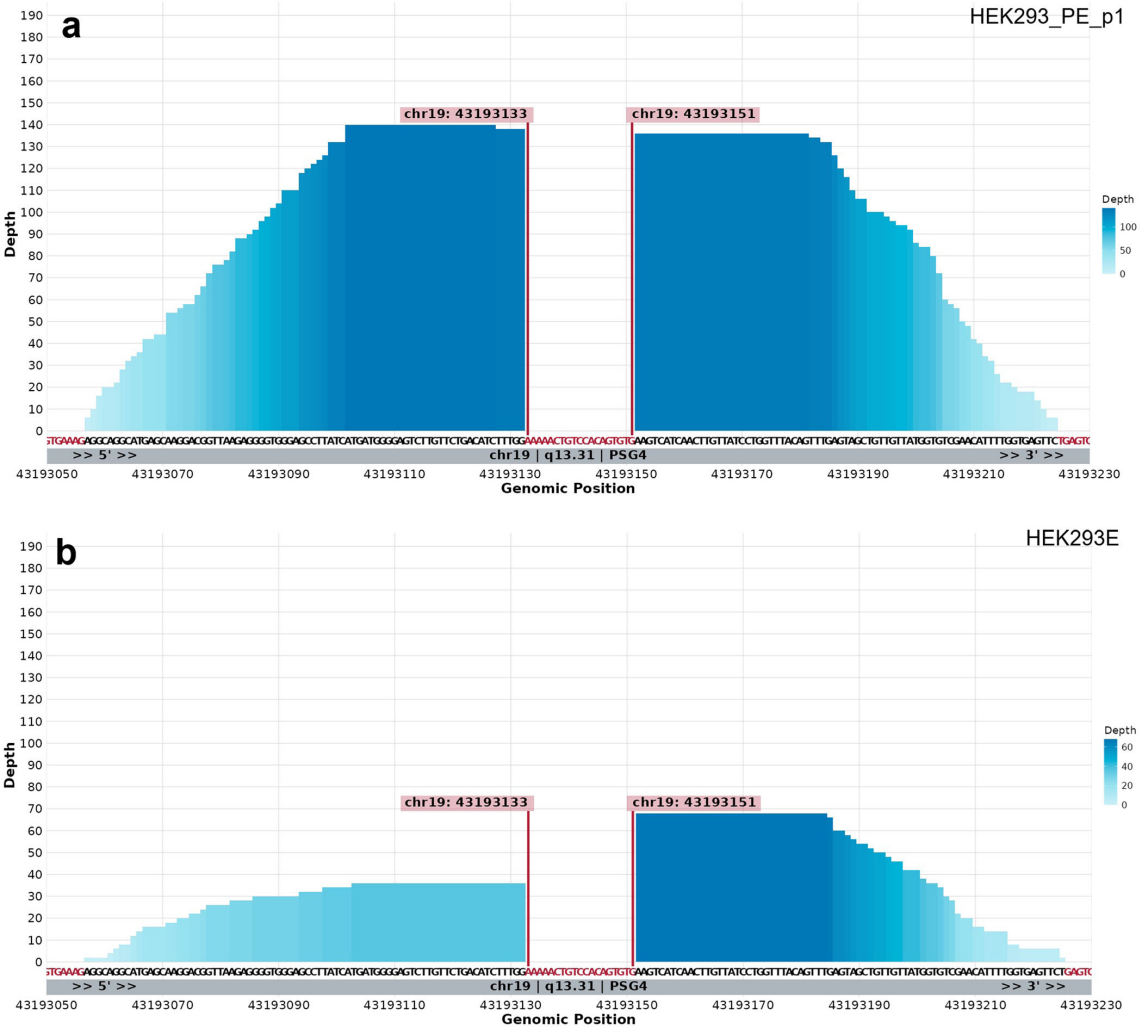
Adenoviral integration site in HEK293 genomes. Depth of covered regions at site of viral integration after realignment of chimeric reads in HEK293_PE_p1 (a) and HEK293E (b). The red‐marked sequence indicates a 19 bp deletion at the site of integration, while the red flags denote the start and end points of the integration. The location is specified below, on the long arm of chromosome 19 at band 13.31, within the PSG4 gene. Differences in depth of coverage between a and b reflect variations in sequencing depth between the two compared datasets.

To further investigate this adenovirus integration site, chimeric reads containing both viral and human sequences were realigned to the human reference genome. The integration was located on the long arm of chromosome 19 at band 13.31 (q13.31), within the pregnancy‐specific beta‐1‐glycoprotein 4 gene (PSG4, Figure 4) and was confirmed for all analyzed cell lines (Supporting Information Figures S16 to S19). Consistent with the originally reported adenovirus 5 integration sequence, a 19 base pair deletion in PSG4 was confirmed, further supporting a highly conserved and stable structure at the locus of integrated adenoviral genes (Louis et al. 1997).

Furthermore, the integrated 4 kb segment of adenovirus 5 was examined for genomic mutations across all cell lines. This highly conserved region showed no SNPs or indels, with the exception of a single SNP in HEK293E within the open reading frame of E1A, resulting in a C‐to‐G substitution. This variant is predicted to cause a missense mutation, leading to a leucine‐to‐valine substitution with no to minimal predicted impact on E1A protein function. Collectively, the stable copy number of the adenoviral region, the conserved integration site, and the near‐complete absence of genetic variation in this region underscore its importance in maintaining any type of HEK293 cells.

## 3 Discussion

Scalable, serum‐free suspension cultures are central for efficient bioprocessing, particularly in the context of biopharmaceuticals, necessitating the availability of fast‐growing, suspension‐adapted HEK293 cell lines, along with a fundamental understanding of their behavior. In this study, we successfully transitioned adherent growing HEK293 cells directly to suspension growth, using four different available serum‐free media formulations. This approach not only maintained high cell viability, consistent with previous findings (Jang et al. 2022; Cervera et al. 2011), but also achieved adaptation within a timeframe comparable to highly optimized protocols for CHO cell adaptation involving gradual serum reduction and nutrient supplementation (Wu 2021). Despite the use of anti‐clumping agents, we observed slightly varied morphological behavior. Specifically in cell aggregation tendencies, dependent on the chosen medium which may influence transfectability and productivity. Media compositions optimized for high transfectability and/or viral vector production (PE, BalCD and F17) also supported high cell growth of up to 9 × 10^6^ cell. mL^−1^, with a doubling time of approximately 30 h. Notably, these cell densities exceed those reported for other serum‐free adaptation protocols and are comparable to those of adapted cultures supplemented with FBS (Jang et al. 2022; Cervera et al. 2011).

To investigate potential correlations between genotype variations and specific phenotypes in HEK293‐derived cell lines, in particular with a focus on genome changes that follow from the adaptation process, we analyzed whole‐genome sequences of the original adherent HEK293 cell line, the newly generated serum‐free suspension cell lines, and previously published whole‐genome sequences of several HEK293 lineages (Malm et al. 2020). Similar to observations in Chinese hamster ovary (CHO) cell lines, large structural variants accounted for the highest degree of diversity across all samples. While they occur less frequently than small variants, the relatively large number of unique large SVs in each sample reflect their contribution to the overall genomic diversity (Figure 2c). In contrast to CHO, where translocations are common, deletions represented the predominant type of structural variation in HEK293. While these large‐scale alterations contribute to genetic diversity, the majority of genomic divergence between HEK293 cell lines appears to arise from the accumulation of small variants (SNPs and indels) over time. The genomic divergence observed in CHO cell lines, where small variants can serve as markers to trace monoclonality and population dynamics, is similarly reflected in HEK293‐derived lines (Kuhn et al. 2020). Due to the short time of their existence, the here established suspension adapted cell lines showed a high degree of shared small genetic variants with their parental adherent HEK293 cells, as reflected in the total number of variants they have in common (Figure 2a and b). In 12 out of the 20 largest SNP combinations (Figure 2a) and 7 out of the 20 largest indel combinations (Figure 2b), the parental HEK293_adherent cells and all directly derived suspension‐adapted cell lines (HEK293_PE_p1, HEK293_PE_p2, HEK293_CD293, HEK293_BalCD, and HEK293_F17) shared the complete set of small variants, indicating a close genetic relationship. In contrast, cell lines that underwent genetic modifications or extended clonal selection such as HEK293_6E, HEK293T, HEK293E, and HEK293H, displayed increasingly distinct variant profiles (Figure 2a to c), as indicated by the smaller number of variants shared with other cell lines and the large sets of unique small variants in each individual cell line (Figure 2a,b). Among these, HEK293E was revealed as the most divergent, due to the smallest overall number of shared variants across all samples, consistent with previous reports (Malm et al. 2020). These findings suggests, that as in CHO cells, the accumulation of small variants in HEK293 are influenced by multiple factors, such as the clonal origin, applied evolutionary pressures, genetic manipulation and/or selection and, in particular time in culture (Feichtinger et al. 2016).

Gene ontology enrichment analysis of three distinct categories of variants, selected based on their presence in different combinations of cell lines, was performed to evaluate their potential biological implications. Not surprisingly, common core mutations revealed significant enrichment in terms associated with cytoskeleton organization, extracellular structure organization, and cell–cell adhesion (Supporting Figure 2 and Supporting Information Table 3). In contrast, analysis of common large rearrangements identified terms related to neuronal development, synaptic membrane organization, and neural connectivity (Supporting Information Figure S3 and Table S4). These processes are all associated with extracellular matrix organization, cellular structure, morphogenesis or cell‐cell connectivity, either in a general cellular context or specifically within neuronal‐like tissues (Gene Ontology 2023; Binns et al. 2009). Considering the neuronal properties of HEK293 cells and their proposed origin from an embryonic adrenal gland precursor (Stepanenko and Dmitrenko 2015) it is conceivable that this cell type was particularly susceptible to large‐scale genomic alterations in these regions during or following adenoviral transformation (Shaw et al. 2002).

Unsurprisingly, enrichment analysis of the third variant category, suspension‐associated variants, identified 45 significantly enriched genes, all linked to the GO term of homophilic cell adhesion via plasma membrane adhesion molecules and, with slightly lowered significance thresholds cell‐cell connectivity, adhesion and signal transduction as may be expected of suspension adapted cells deprived of anchorage.

However, it must be noted that investigation of two additional variant categories, defined by either strict association with suspension conditions or with the direct‐adaptation process, did not identify any significantly enriched mutations related to specific biological terms (Supporting Information S1). The first category focused on variants absent in all adherent cell lines (HEK293, HEK293_adherent, HEK293T, and HEK293T) but present in all suspension cell lines (HEK293_PE_p1, HEK293_PE_p2, HEK293_CD293, HEK293_BalCD, HEK293_F17, HEK293_6E, HEK293H, HEK293F, and HEK293Freestyle). The second category included variants absent in the parental adherent cell line (HEK293_adherent) but present in all directly adapted suspension cell lines (HEK293_PE_p1, HEK293_PE_p2, HEK293_CD293, HEK293_BalCD, HEK293_F17). While the analysis of suspension‐associated variants (absent in both parental adherent cell lines, but present in one or more suspension cell lines) suggested potentially affected cell‐adhesion proteins as part of a long‐term adaptation process, the evaluation of short‐term adaptation (8 weeks), following a direct‐adaptation protocol without intermediate steps, could not reveal any significantly enriched functional implications. Analysis of genome‐wide gene copy number ratios revealed alterations across large chromosomal regions in several cell lines, indicating gains and losses of chromosome segments or variation in chromosome numbers over time in culture, as previously reported for HEK293 cells and similarly to what has been described in CHO (Bylund et al. 2004; Binz et al. 2019; Derouazi et al. 2006; Vcelar et al. 2018). While these broader genomic changes reflect continuously ongoing divergence across the genomes, the integration site of adenoviral genes, previously described as a genomic safe harbor, remained preserved in all analyzed HEK293 cell lines (Shin et al. 2020). Furthermore, variant analysis of the integrated adenoviral sequence revealed only a single SNP in HEK293E, potentially leading to an amino acid substitution within the E1A viral gene, with predicted no to mild effect on the gene function. This, together with preserved copy number and chromosomal context of the adenoviral genes (Figure 3f,g) suggest a high importance of functional conservation, likely due to their essential roles in host cell cycle regulation (Moran and Mathews 1987; Flint and Shenk 1997), apoptosis suppression (Cuconati and White 2002), and modulation of innate immune responses (Reich et al. 1988; Bhattacharya et al. 1996; Zhao et al. 2003). These functions are fundamental for sustaining HEK293 in culture and also essential for their role as helper factors in adeno‐associated viral vector production (Meier et al. 2020; Su et al. 2023; Tan et al. 2021).

While our results underscore genomic divergence among HEK293‐derived cell lines, no direct correlation between specific genetic variants and phenotypic traits can be established from sequencing data alone. Additional epigenetic and transcriptomic analyzes, such as DNA‐methylation profiling, histone modification mapping or RNA sequencing, along with studies that aim to link genotypes to phenotypes, such as genome wide screens, could shed further light into gene activity, response and dosage effects that may underlie phenotypic differences relevant to production performance.

## 4 Material and Methods

### 4.1 Adherent Cell Culture

Adherent HEK293 cells obtained from ATCC (ATCC‐CRL‐1573) were maintained in tissue culture flasks (Greiner) using Dulbecco's Modified Eagle Medium (DMEM, Gibco, Thermo Fisher Scientific) supplemented with 5% Fetal Bovine Serum (FBS, Sigma‐Aldrich) and 4 mM l‐glutamine (Sigma‐Aldrich), according to the recommended working volumes. Cells were incubated at 37°C in a humidified atmosphere with 5% CO_2_ in a HERAcell 150i incubator (Thermo Fisher Scientific). When cultures reached 70‐80% confluency, they were subcultured to 10% confluency, using an enzymatic trypsin solution (0.05% Trypsin‐EDTA, Gibco, Thermo Fisher Scientific) detachment, according to recommendations.

### 4.2 Adaptation of Adherent HEK293 Cells to Serum‐Free Suspension

Expanded adherent cultures at approximately 80% confluence were applied to a direct adaptation process. For this, viable cell density was measured using the trypan blue exclusion method in an automated ViCell XR (Beckman Colter) machine. Cells were enzymatically dissociated, harvested by centrifugation (300 g, 5 min at room temperature) and washed in PBS. Next, cells were resuspended in the corresponding media formulation for adaptation, namely HyClone peak expression medium (Cytiva, named PE), CD293 medium (Gibco, named CD293), BalanCD HEK293 medium (Fujifilm, named BalCD) and Freestyle F17 medium (Gibco, named F17), targeting a seeding density of 1 × 106 cell. mL^−1^ in biological triplicates. All media except PE were supplemented with 4 mM l‐Glutamine (Sigma‐Aldrich), 0.1% Poloxamer 188 (Gibco, Thermo Fisher Scientific) and 0.2% Anti‐Clumping‐Agent (Gibco, Thermo Fisher Scientific). Suspension cells were maintained in 50 mL TubeSpin Bioreactors (TPP) using a working volume of 15 mL. Cultures were cultivated at 37°C at 80% humidity and 5% CO_2_ under 220 rpm of shaking in a Climo‐Shaker ISF4‐X (Kuhner).

Cells were subcultured twice per week at a seeding density of 4 × 10⁵ cell. mL^−1^ until specific growth rates stabilized. After an 8‐week adaptation period, cell banks were prepared using 2 ×107 cells, preserved in a mixture of the respective medium and 10% DMSO (Sigma‐Aldrich) and then stored in liquid nitrogen.

### 4.3 DNA Preparation, Library Preparation and Sequencing

DNA samples were collected at the start of the direct adaptation process from expanded adherent cultures and from each suspension‐adapted cell line prior cell banking. Total genomic DNA was harvested from approximately 3 × 10⁶ cells per sample via centrifugation (300 g, 5 min, room temperature) and isolated using the DNeasy Blood and Tissue Kit (Qiagen), following the manufacturer's guidelines. The concentration of purified genomic DNA was measured using a NanoDrop One C spectrophotometer (Thermo Scientific). Genomic DNA was prepared for sequencing using an enzymatic DNA shearing kit. Library Preparation and Whole‐genome sequencing was subsequently performed on a NovaSeq X platform (Illumina) by the Next Generation Sequencing Facility at the Vienna BioCenter Core Facilities (VBCF). Sequencing was conducted in paired‐end mode with a read length of 150 base pairs. On average, in‐house generated samples yielded approximately 1.6 billion reads per sample, corresponding to an estimated mean coverage depth of ~80× over the human reference genome, while publicly available samples from Malm et al. achieved an average coverage of ~40× (Supporting Information Table S8).

### 4.4 Data Processing

A reproducible and traceable data analysis pipeline was developed using the workflow management system Snakemake (Mölder et al. 2021) in combination with the Conda package manager. This semi‐automated workflow includes all utilized tools, their versions, and configurations for data processing and is available at the author's github repository (https://github.com/NBorthLab/HEK293_genomes).

In detail, quality control was performed using FastQC (Andrews 2010) to assess read quality, while alignment quality was evaluated with the Genome Analysis Toolkit (GATK) (McKenna et al. 2010; van der Auwera and O'Connor 2020) and BEDTools (Quinlan and Hall 2010). MultiQC was then used to gather and summarize quality metrics (Ewels et al. 2016). Next, adapter sequences in raw reads were removed with Trimmomatic (Bolger et al. 2014), using TruSeq. 3‐PE‐2 adapter sequence templates. Genome sequencing reads were aligned with BWA‐MEM (Li and Durbin 2010; Li 2013) against a customized reference genome, which combined the human reference genome (hg38, UCSC) (Perez et al. 2025) and the human adenovirus 5 reference (RefSeq number AC_000008.1) referred to as HAdV.5 (O'Leary et al. 2016). Raw alignments were sorted with SAMtools (Li et al. 2009) and deduplicated using MarkDuplicates from the GATK suite. Small genomic variants were identified using GATK HaplotypeCaller and subsequently categorized and filtered with SelectVariants and VariantFiltration from the GATK bundle (McKenna et al. 2010; van der Auwera and O'Connor 2020). Structural rearrangements were detected with Manta (Chen et al. 2016) and filtered using SURVIVOR (Jeffares et al. 2017), applying a minimum length threshold of 300 base pairs (Supporting Information Table S9). Functional annotation of both small and large variants was performed with SnpEff using the hg38 reference (Cingolani et al. 2012). Copy number alterations were evaluated and visualized with CNVkit using the flat reference option, assuming a diploid copy number across the reference genome (Talevich et al. 2016). Comparative analysis of small and structural variants was conducted in R (R Core Team Foundation for Statistical Computing 2013) using custom scripts. Variant evaluation was performed with the vcfR (Knaus and Grünwald 2017), VariantAnnotation (Obenchain et al. 2014), and Biostrings (Pagès 2019) packages, while visualization was generated using UpSetR (Gehlenborg and Conway 2019) and ggplot (Wickham 2011) from the Tidyverse suite (Wickham et al. 2019). Preparation of variant categories before gene ontology enrichment analysis was performed using custom scripts or manual selection. GO term enrichment analysis was conducted with the clusterProfiler package (Yu et al. 2012). Unless otherwise indicated in the provided source code (https://github.com/NBorthLab/HEK293_genomes), all tools were used in their default mode.

## Author Contributions

**Georg Smesnik:** conceptualization, writing – original draft, formal analysis, investigation, visualization. **Nikolaus Virgolini:** conceptualization, writing – review and editing, validation. **Maria Toth:** investigation. **Astrid Dürauer:** conceptualization, funding acquisition, project administration, writing – review and editing. **Nicole Borth:** conceptualization, supervision, writing – original draft, writing – review and editing.

## Conflicts of Interest

The authors declare no conflicts of interest.

## Supporting information

supplementary_material.

supplementary_table_1_SNP_rates.

supplementary_table_2_indel_rates.

supplementary_table_3_GOterm_common_core_mutations.

supplementary_table_4_GOterm_common_large_SV.

supplementary_table_5_GOterm_suspension_associated_default.

supplementary_table_6_Description_45_enriched_genes.

supplementary_table_7_GOterm_suspension_associated_reduced.

supplementary_table_8_Alignment_metrics.

supplementary_table_9_SV_size_exclusion.

## Data Availability

The data that support the findings of this study are openly available in European Nucleotide Archive at https://www.ebi.ac.uk/ena/browser/home, reference number PRJEB86622.
