# Supplementary material for: Comparative Analysis of HEK293 Genomic Variability
Source: Biotechnol Bioeng. 2025 Nov 10;123(2):436–48. doi: 10.1002/bit.70105 (PMC12779178; doi:10.1002/bit.70105)
Supplement: Supplementary file 1 — supplementary_material. [file BIT-123-436-s001.docx]

# Supplementary Material

Content of supplementary material

1. Variant appearance rates during direct-adaptation
2. Gene ontology enrichment analysis of genomic variants
3. Genome-wide copy number alteration analysis
4. Copy number alteration analysis of integrated adenoviral sequences
5. Detection of human adenovirus 5 integration site
6. Additional information

## Variant appearance rates during direct-adaptation

The presented Venn diagrams illustrate overlapping variants from pairwise comparisons of the indicated samples. All types of variants, SNPs, indels and large structural rearrangements (length > 300 bp) were considered. The number below each sample name represents the total variants, relative to the human reference genome, identified in that sample combination. HEK293_6E was included as a suspension reference but is not directly derived from HEK293_adherent

.
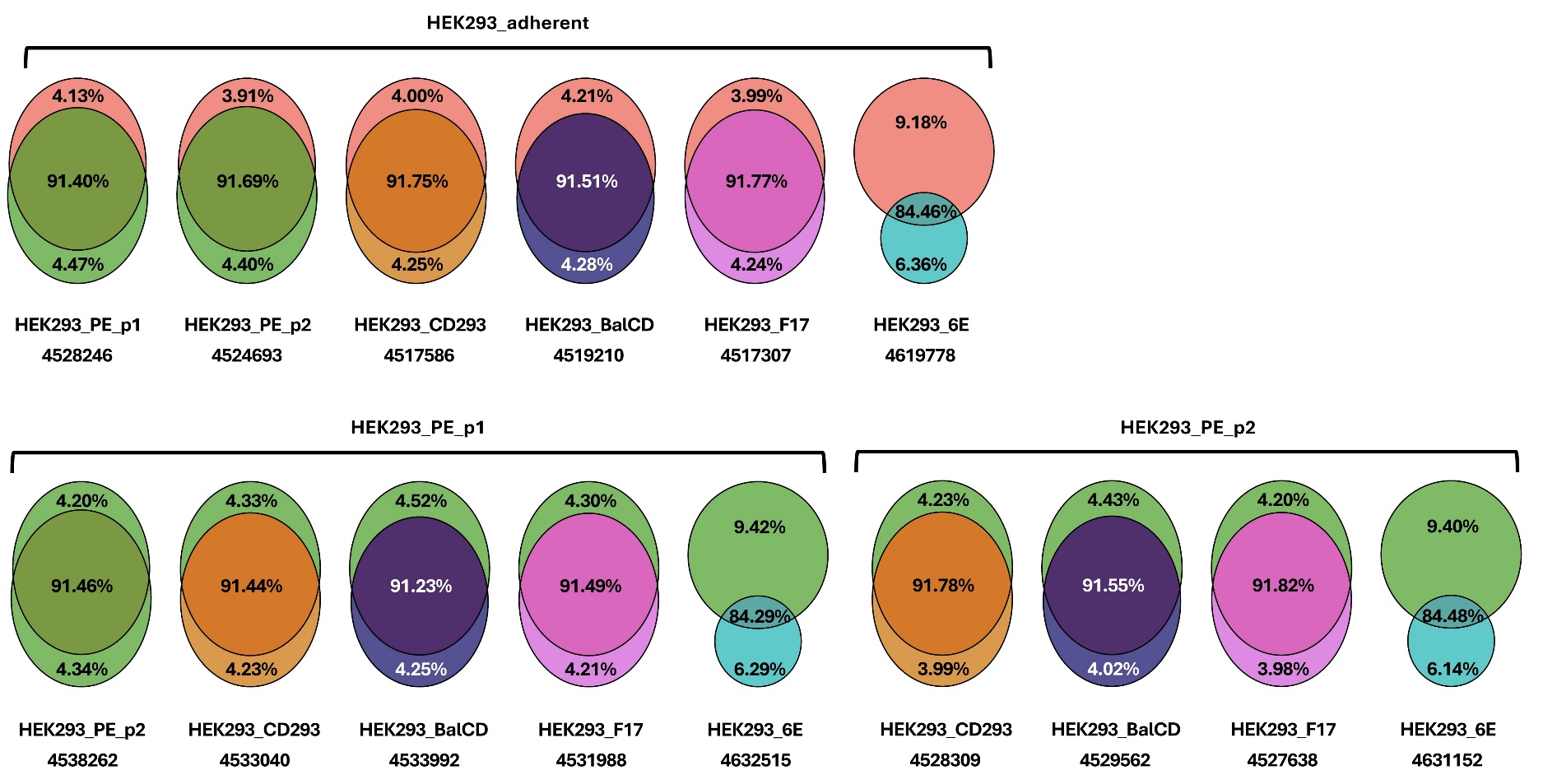


**Supplementary Figure 1**: Venn diagrams of pairwise variant comparisons between the indicated samples. Each diagram shows shared variants (center) and variants unique to each sample (top and bottom). The number below each sample name indicates the total variants, relative to the human reference genome, detected in the corresponding sample combination

## Gene ontology enrichment analysis of genomic variants

Genomic variants were analyzed for gene ontology (GO) enrichment to assess their potential biological impact. Depending on the research objective, specific subsets of variants were selected based on defined attributes prior to GO term enrichment analysis. For all variant types (SNP, indel, SV), only those with a predicted high or moderate impact, as annotated using snpEff, were included. High impact variants are most likely disruptive due to nonsense mutations (stop gains, frameshifts, splice donor/acceptor changes), while moderate impact variants might lead to functional changes due to missense mutations (amino acid substitutions) or in-frame indels. Both homozygote and heterozygote variants were considered.

Three specific objectives were addressed for the analysis:

1. **Common core mutations:** Variants (SNP, indel, SV) shared by all observed 13 samples
2. **Common large structural rearrangements:** Structural variants larger than 300 bp, shared across all 13 samples
3. **Suspension associated variants:** Variants (SNP, indel, SV) absent in both parental adherent cell lines (HEK293, HEK293_adherent) but present in one or more suspension cell lines (HEK293_PE_p1, HEK293_PE_p2, HEK293_CD293, HEK293_BalCD, HEK293_F17, HEK293_6E, HEK293H, HEK293F, HEK293Freestyle)

Further objectives addressed for GO term analyses did not reveal any significantly enriched gene sets:

1. **Strictly common suspension associated variants:** Variants (SNP, indel, SV) absent in all adherent cell lines (HEK293, HEK293_adherent, HEK293E, HEK293T) and present in all suspension cell lines (see ii)
2. **Strictly direct-adaptation associated variants:** Variants (SNP, indel, SV) absent in the internal parental adherent cell line HEK293_adherent, and present in all directly suspension-adapted cell lines (HEK293_PE_p1, HEK293_PE_p2, HEK293_CD293, HEK293_BalCD, HEK293_F17)

Gene symbols of affected genes were extracted from the variant lists and subjected to GO term enrichment analysis, enabling the identification of biological processes or cellular components potentially impacted by the corresponding genomic mutation.

Detailed numerical results and significance thresholds are provided in supplementary tables 5-8. Supplementary table 9 includes a detailed description of genes associated with GO:0007156 (homophilic cell adhesion via plasma membrane adhesion molecules), identified as enriched in the analysis for suspension associated variants (ii).

### Common core mutations

All types (SNP, indel, SV) with high or moderate impact, shared across all 13 samples


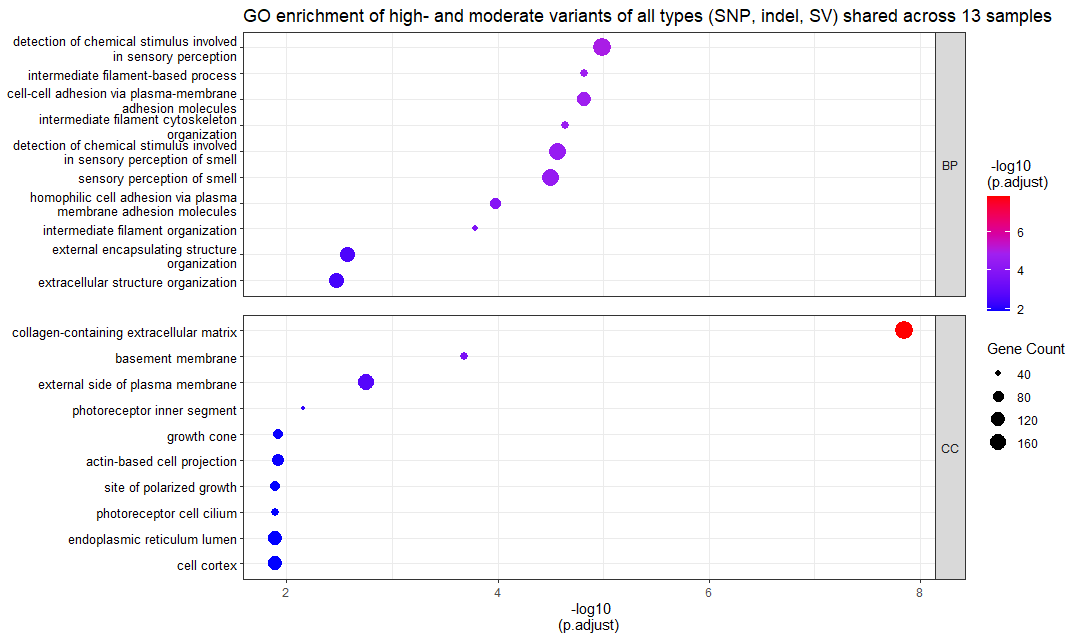


**Supplementary Figure 2**: Gene Ontology (GO) term enrichment analysis of selected genomic variants. Dot size represents the number of enriched genes associated with each GO term, while dot color reflects the significance of enrichment, expressed as the negative log10 of the adjusted p-value, with red indicating high significance and blue indicating low significance. GO terms are categorized into biological processes (BP; upper panel) and cellular components (CC; lower panel). GO enrichment analysis settings: pAdjustMethod = BH (Benjamini & Hochberg), pvalueCutoff = 0.05, qvalueCutoff = 0.2. Top 10 significant GO terms are shown here, while the complete enrichment analysis is provided in Supplementary table 5.

### Common large structural rearrangements

Large structural variants (length > 300 bp) and shared across all 13 samples.


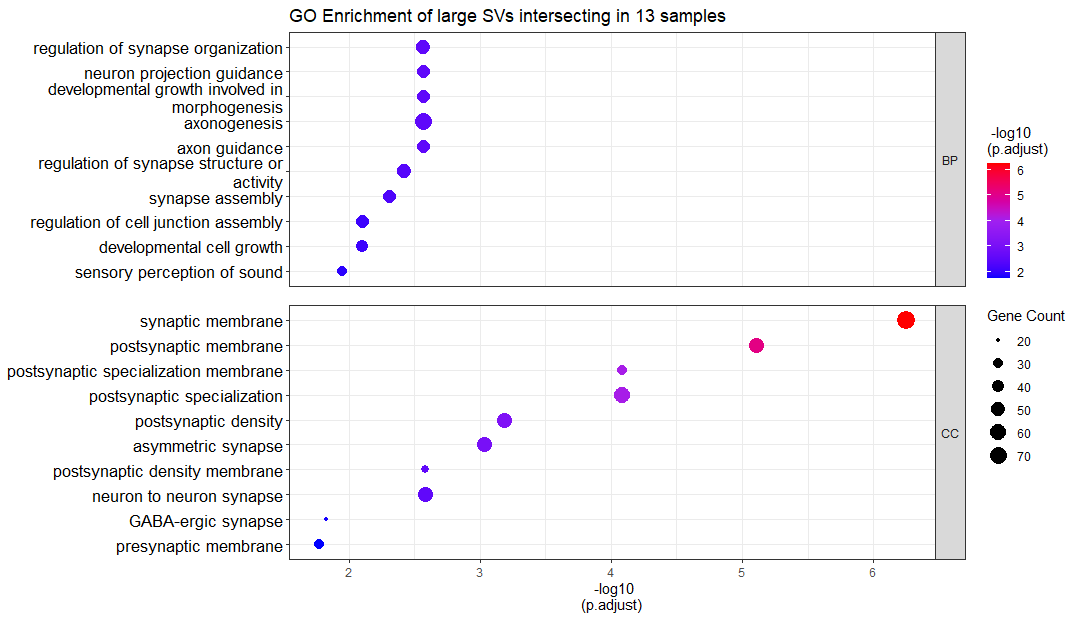


**Supplementary Figure 3**: Gene Ontology (GO) term enrichment analysis of selected genomic variants. Dot size represents the number of enriched genes associated with each GO term, while dot color reflects the significance of enrichment, expressed as the negative log10 of the adjusted p-value, with red indicating high significance and blue indicating low significance. GO terms are categorized into biological processes (BP; upper panel) and cellular components (CC; lower panel). GO enrichment analysis settings: pAdjustMethod = BH (Benjamini & Hochberg), pvalueCutoff = 0.05, qvalueCutoff = 0.2. Top 10 significant GO terms are shown here, while the complete enrichment analysis is provided in Supplementary table 6.

### Suspension associated variants

All types (SNP, indel, SV) with high or moderate impact absent in both adherent, parental cell lines (HEK293, HEK293_adherent) and present in one or more suspension cell lines (reduced significance thresholds).


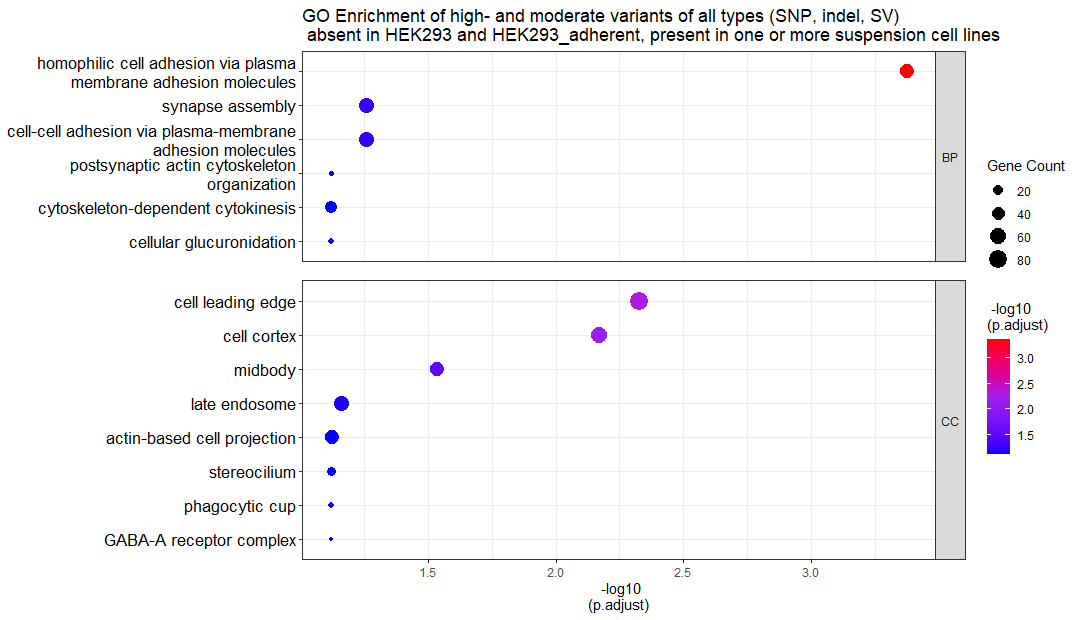


**Supplementary Figure 4**: Gene Ontology (GO) term enrichment analysis of selected genomic variants. Dot size represents the number of enriched genes associated with each GO term, while dot color reflects the significance of enrichment, expressed as the negative log10 of the adjusted p-value, with red indicating high significance and blue indicating low significance. GO terms are categorized into biological processes (BP; upper panel) and cellular components (CC; lower panel). GO enrichment analysis settings: pAdjustMethod = BH (Benjamini & Hochberg), pvalueCutoff = 0.08, qvalueCutoff = 0.3 (reduced significance threshold). Top 10 significant GO terms are shown here, while the complete enrichment analysis is provided in Supplementary table 7 and 8.

## Genome-wide copy number alteration analysis

The presented copy number alteration analysis from sequencing data was performed using the flat-reference option of the CNVkit toolkit. This option assumes a baseline of normal, diploid copy number across the genome (log2 = 0.0), allowing the estimation of gains and losses in genomic regions relative to the expected diploid state. Copy number estimation is based on regional deviations from the expected depth of coverage.

To identify regional differences in copy number, the genome is segmented into small sections of defined size, called bins. Each bin represents a certain stretch of DNA (i.e. 200 bp) and the number of sequencing reads mapped to each bin is used to calculate the copy number. These bins are shown as individual points on a scatter plot, arranged by their genomic position across the analyzed region, or the entire genome. Depending on the chromosomal length, the number of bins varies per chromosome, resulting in sections of different width in the final plot, proportional to the size of each chromosome. Trends in neighboring bins indicating larger-scale amplifications or deletions are marked by red horizontal lines. The y-axis of the scatter plot represents the log2 copy ratio, a scale that reflects fold changes in copy number relative to the expected diploid state. A log2 ratio of 0.0 indicates a normal copy number (two copies), positive values suggest gains, and negative values suggest losses.

The overall resolution of the analysis is determined by the chosen bin size. Smaller bins provide higher resolution but may introduce more variability, while larger bins smooth the signal at the cost of detail. For the presented analysis the recommended bin size for human sequences of 267 bp per bin was chosen.


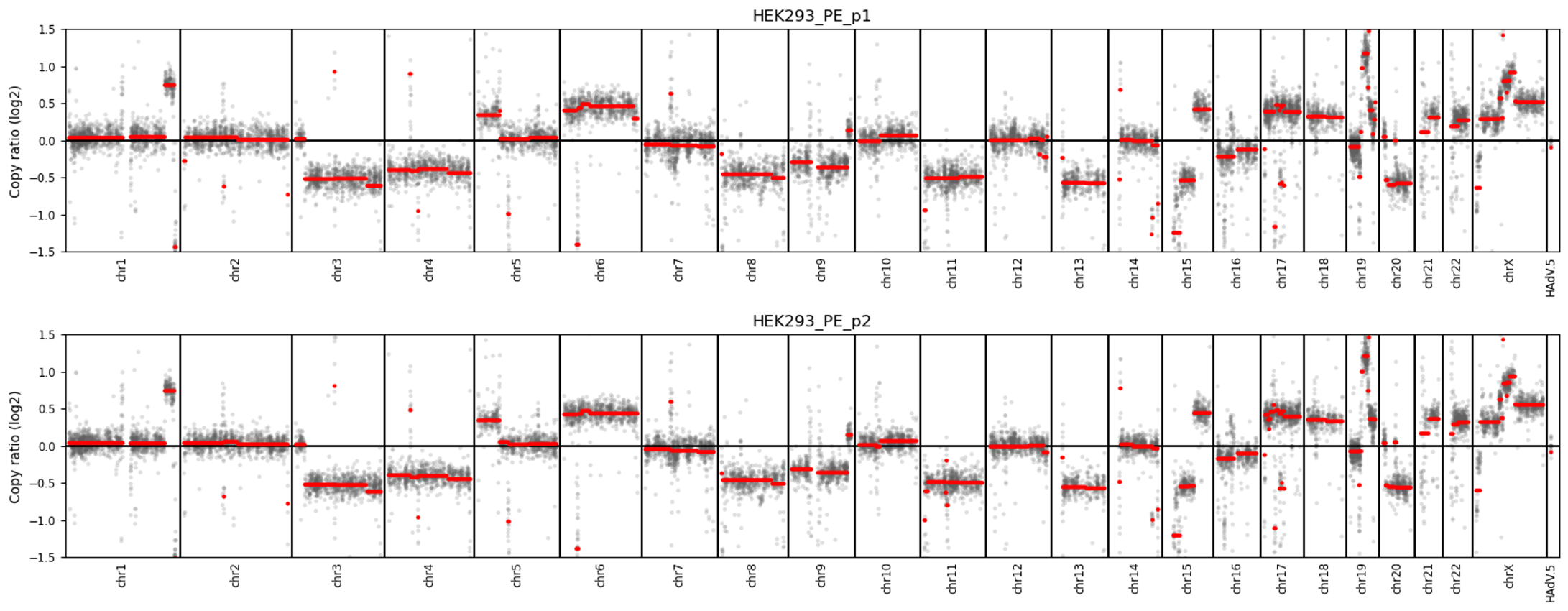


**Supplementary Figure 5**: Genome-wide copy number variation (CNV) analysis of parallel suspension-adapted cell lines HEK293_PE_p1 and HEK293_PE_p2. The scatter plot reveals calculated bins across the entire human genome (chr1-chr22, chrX) and the additional human adenovirus 5 scaffold (HAdV.5). Values are presented on a log2 scale, with 0 corresponding to the expected diploid state, positive values indicate gains, and negative values indicate losses. Trends in copy number alterations are highlighted by red horizontal lines.


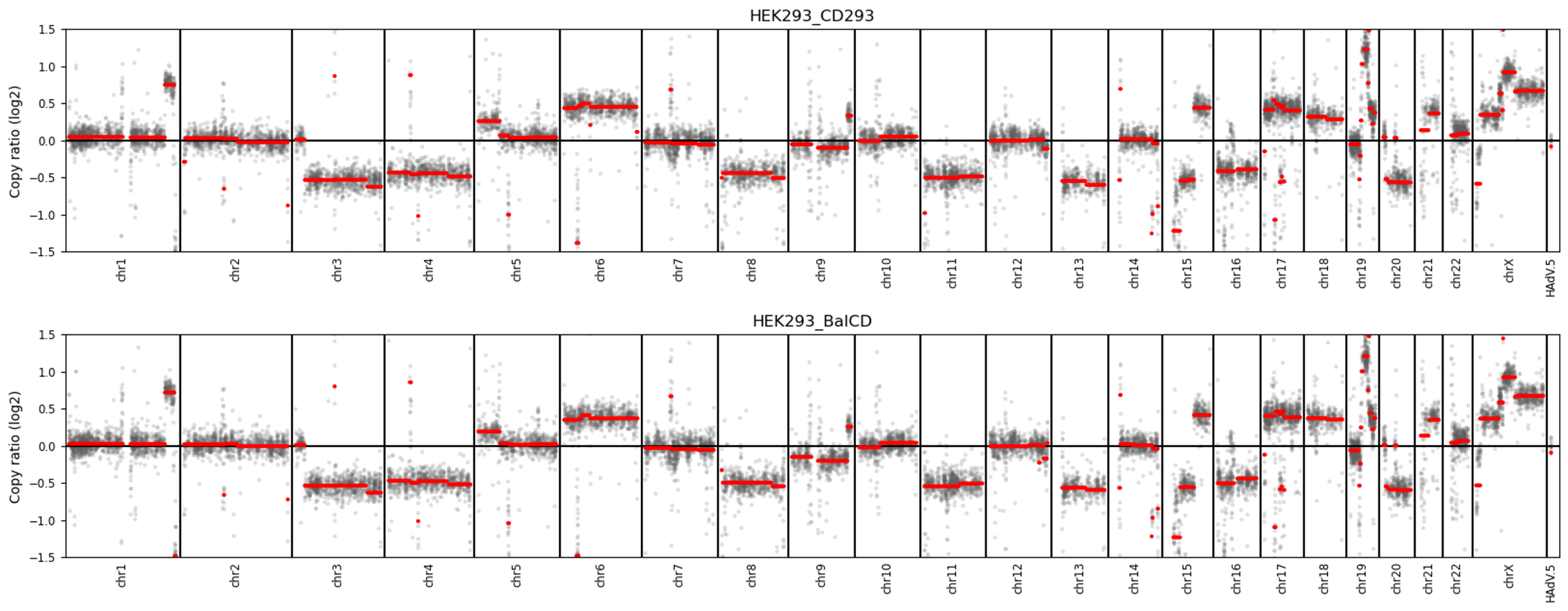


**Supplementary Figure 6**: Genome-wide copy number variation (CNV) analysis of suspension-adapted cell lines HEK293_CD293 and HEK293_BalCD. The scatter plot reveals calculated bins across the entire human genome (chr1-chr22, chrX) and the additional human adenovirus 5 scaffold (HAdV.5). Values are presented on a log2 scale, with 0 corresponding to the expected diploid state, positive values indicate gains, and negative values indicate losses. Trends in copy number alterations are highlighted by red horizontal lines.


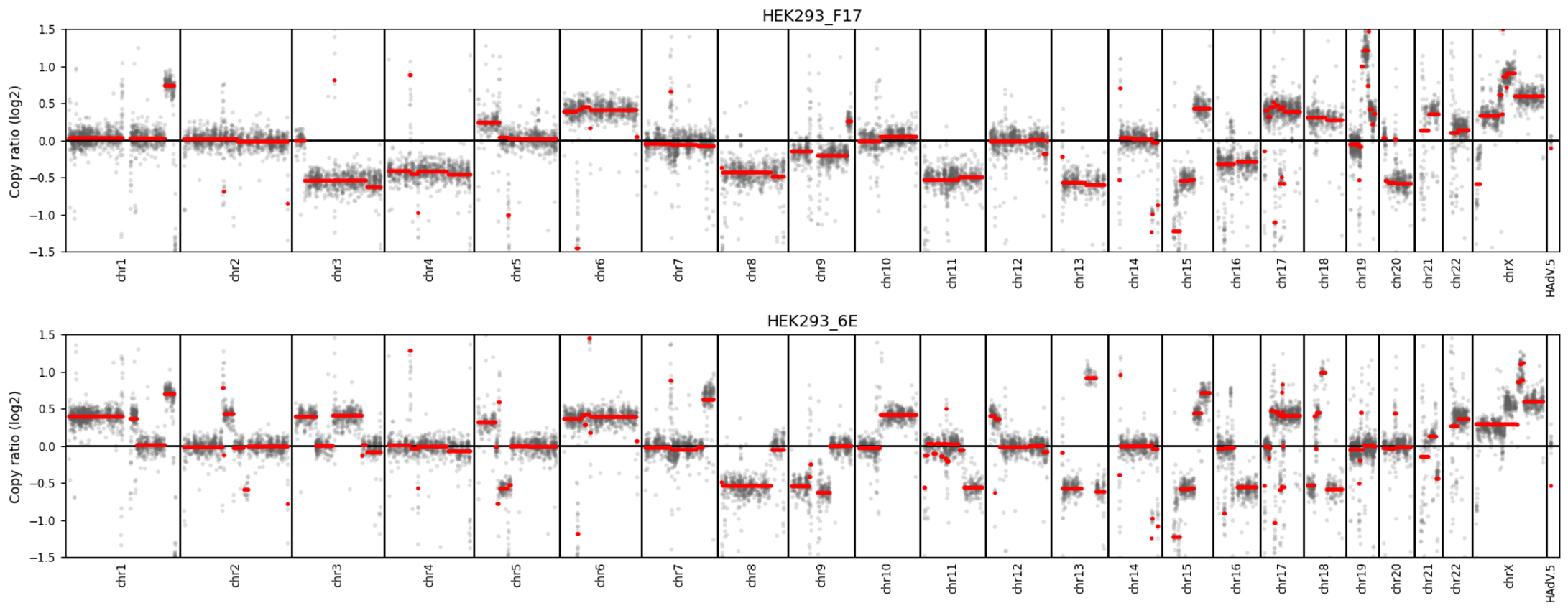


**Supplementary Figure 7**: Genome-wide copy number variation (CNV) analysis of suspension-adapted cell line HEK293_F17 and truncated EBNA1-expressing suspension cell line HEK293_6E. The scatter plot reveals calculated bins across the entire human genome (chr1-chr22, chrX) and the additional human adenovirus 5 scaffold (HAdV.5). Values are presented on a log2 scale, with 0 corresponding to the expected diploid state, positive values indicate gains, and negative values indicate losses. Trends in copy number alterations are highlighted by red horizontal lines.


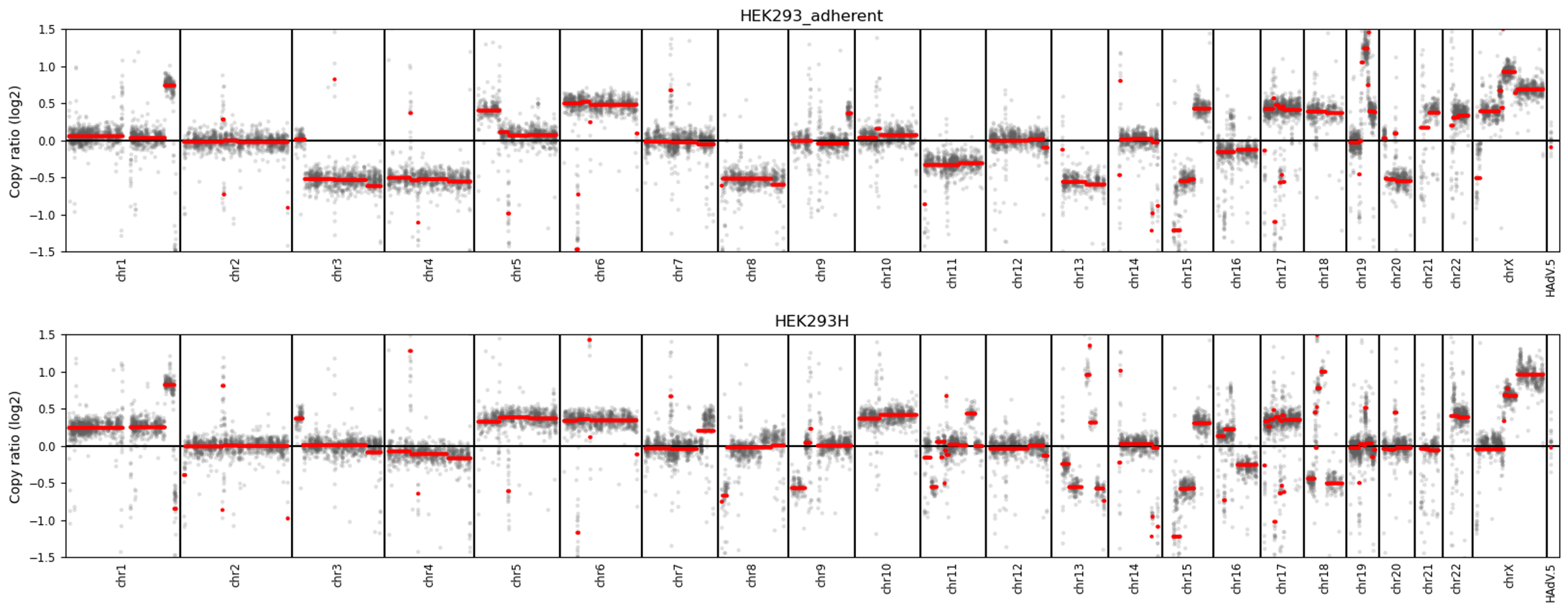


**Supplementary Figure 8**: Genome-wide copy number variation (CNV) analysis of internal parental, adherent cell line HEK293_adherent and clonally selected suspension cell line HEK293H. The scatter plot reveals calculated bins across the entire human genome (chr1-chr22, chrX) and the additional human adenovirus 5 scaffold (HAdV.5). Values are presented on a log2 scale, with 0 corresponding to the expected diploid state, positive values indicate gains, and negative values indicate losses. Trends in copy number alterations are highlighted by red horizontal lines.


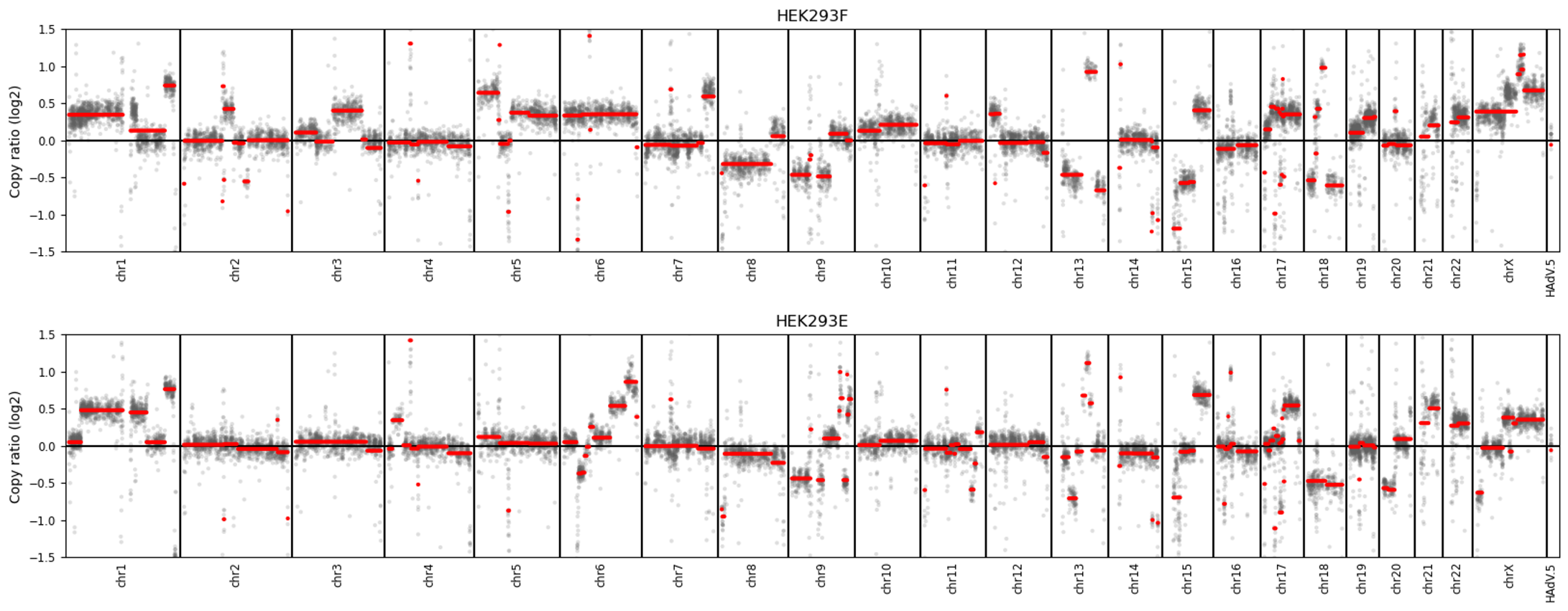


**Supplementary Figure 9**: Genome-wide copy number variation (CNV) analysis of clonally selected suspension cell line HEK293F and EBNA1-expressing adherent cell line HEK293E. The scatter plot reveals calculated bins across the entire human genome (chr1-chr22, chrX) and the additional human adenovirus 5 scaffold (HAdV.5). Values are presented on a log2 scale, with 0 corresponding to the expected diploid state, positive values indicate gains, and negative values indicate losses. Trends in copy number alterations are highlighted by red horizontal lines.


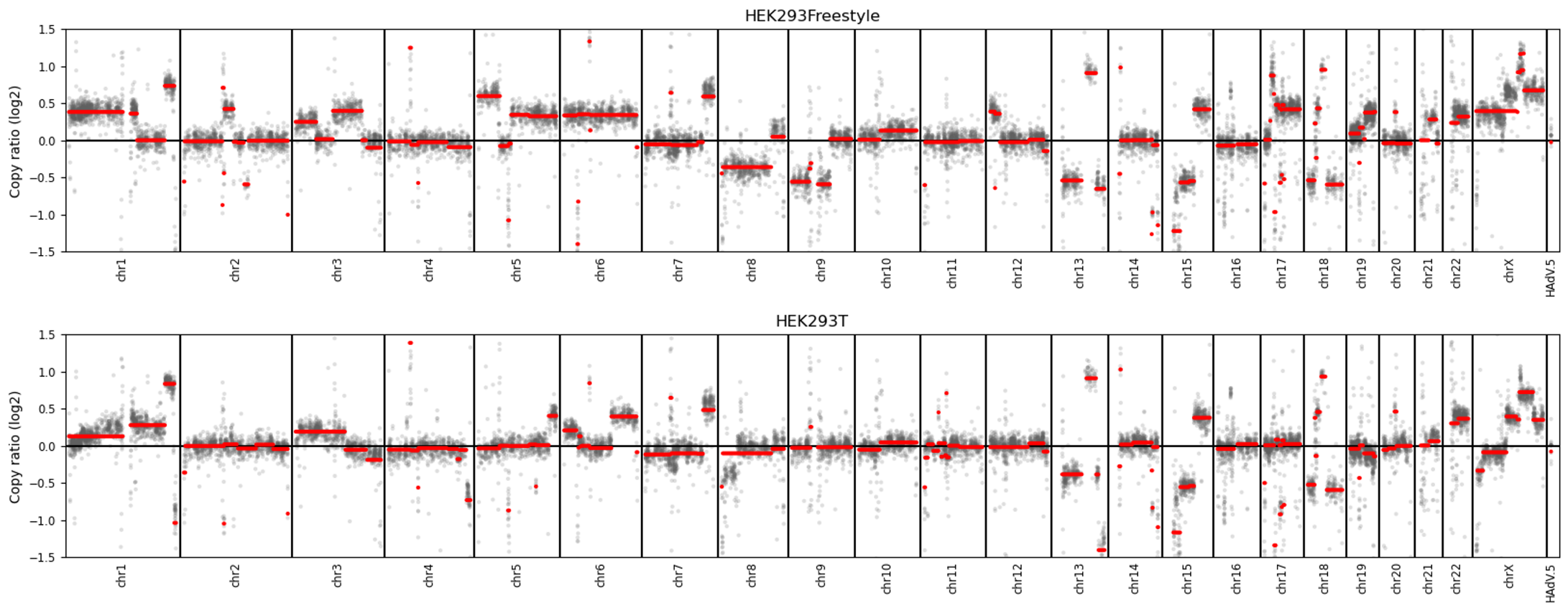


**Supplementary Figure 10**: Genome-wide copy number variation (CNV) analysis of medium adapted suspension cell line HEK293Freestyle and large T-antigen expressing adherent cell line HEK293T. The scatter plot reveals calculated bins across the entire human genome (chr1-chr22, chrX) and the additional human adenovirus 5 scaffold (HAdV.5). Values are presented on a log2 scale, with 0 corresponding to the expected diploid state, positive values indicate gains, and negative values indicate losses. Trends in copy number alterations are highlighted by red horizontal lines.


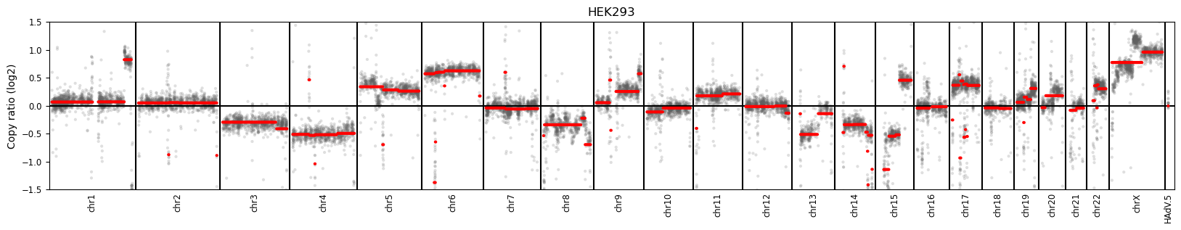


**Supplementary Figure 11**: Genome-wide copy number variation (CNV) analysis of external parental, adherent cell line HEK293. The scatter plot reveals calculated bins across the entire human genome (chr1-chr22, chrX) and the additional human adenovirus 5 scaffold (HAdV.5). Values are presented on a log2 scale, with 0 corresponding to the expected diploid state, positive values indicate gains, and negative values indicate losses. Trends in copy number alterations are highlighted by red horizontal lines.

## Copy number alteration analysis of integrated adenoviral sequences

Presented here are the results of the copy number variation (CNV) analysis for the 4 kb human adenovirus 5 (HAdV.5) segment integrated into the HEK293 genome. This 4344 bp sequence contains open reading frames for four viral genes: early region 1A (E1A), early region 1B (E1B), protein IX (IX), and the intermediate-early transcript IVa2 (IVa2), as well as short, truncated fragments of the inverted terminal repeats (ITRs).

Given the role of these viral elements in maintaining the immortalized phenotype, supporting cell proliferation, and providing advantages for bioproduction applications, the genomic stability of this region is of particular interest.

To enable comparability with the genome-wide CNV analysis, the recommended resolution of 267 bp per bin was maintained, resulting in 16 bins spanning the adenoviral insert. Copy number ratios are plotted on a log2 scale with decimal gradations, enabling the detection of minor variations. The light blue areas indicate the positions of viral genes along the HAdV.5 genome. The overall trend across the 16 bins is indicated by a blue horizontal line.


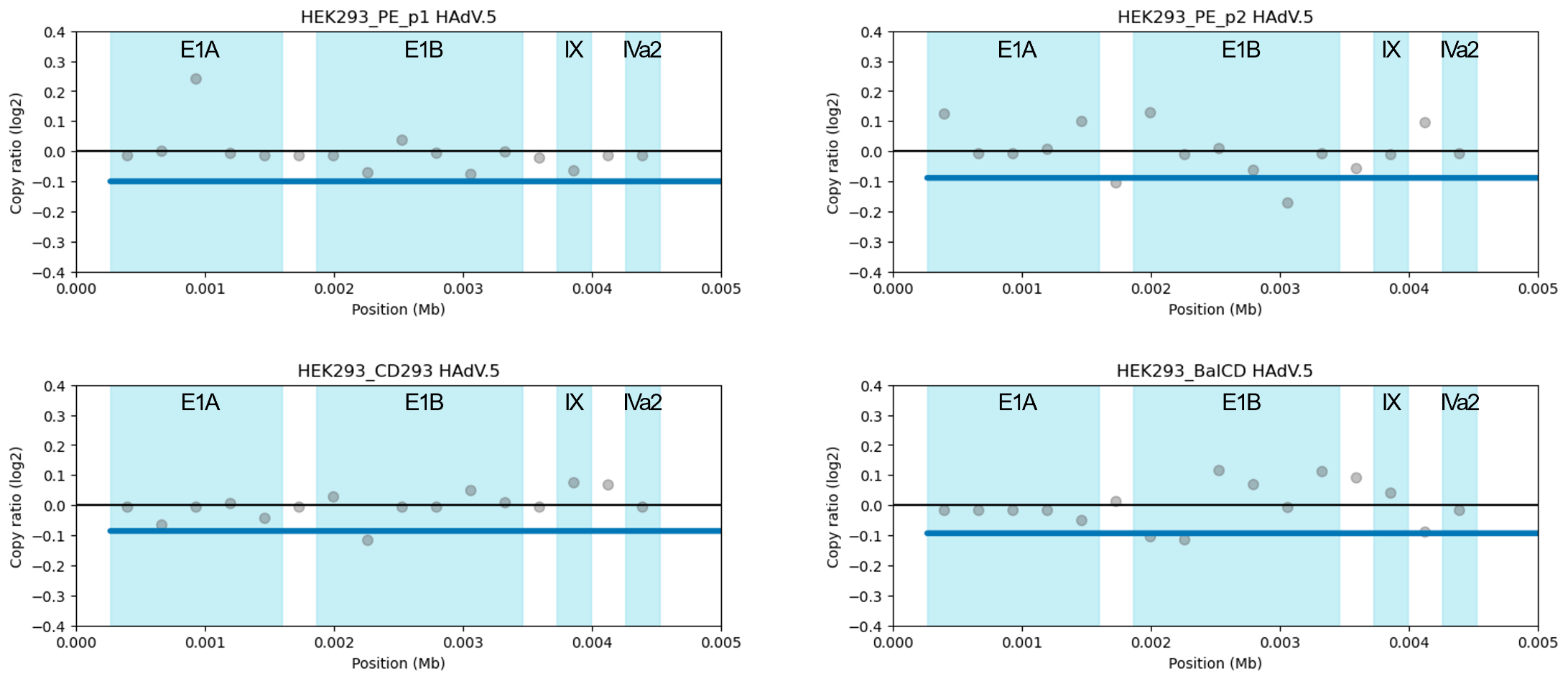


**Supplementary Figure 12**: Copy number variation analysis of integrated adenoviral region for suspension adapted cell lines HEK293_PE_p1, HEK293_PE_p2, HEK293_CD293, HEK293_BalCD. The scatter plot reveals calculated bins across the integrated segment of the human adenovirus 5 genome (HAdV.5). Values are presented on a log2 scale, with 0 corresponding to the diploid state, positive values indicate gains, and negative values indicate losses. Trend in copy number alteration is highlighted by a blue horizontal line. Light blue shaded areas reflect the open reading frames of the corresponding human adenoviral genes.


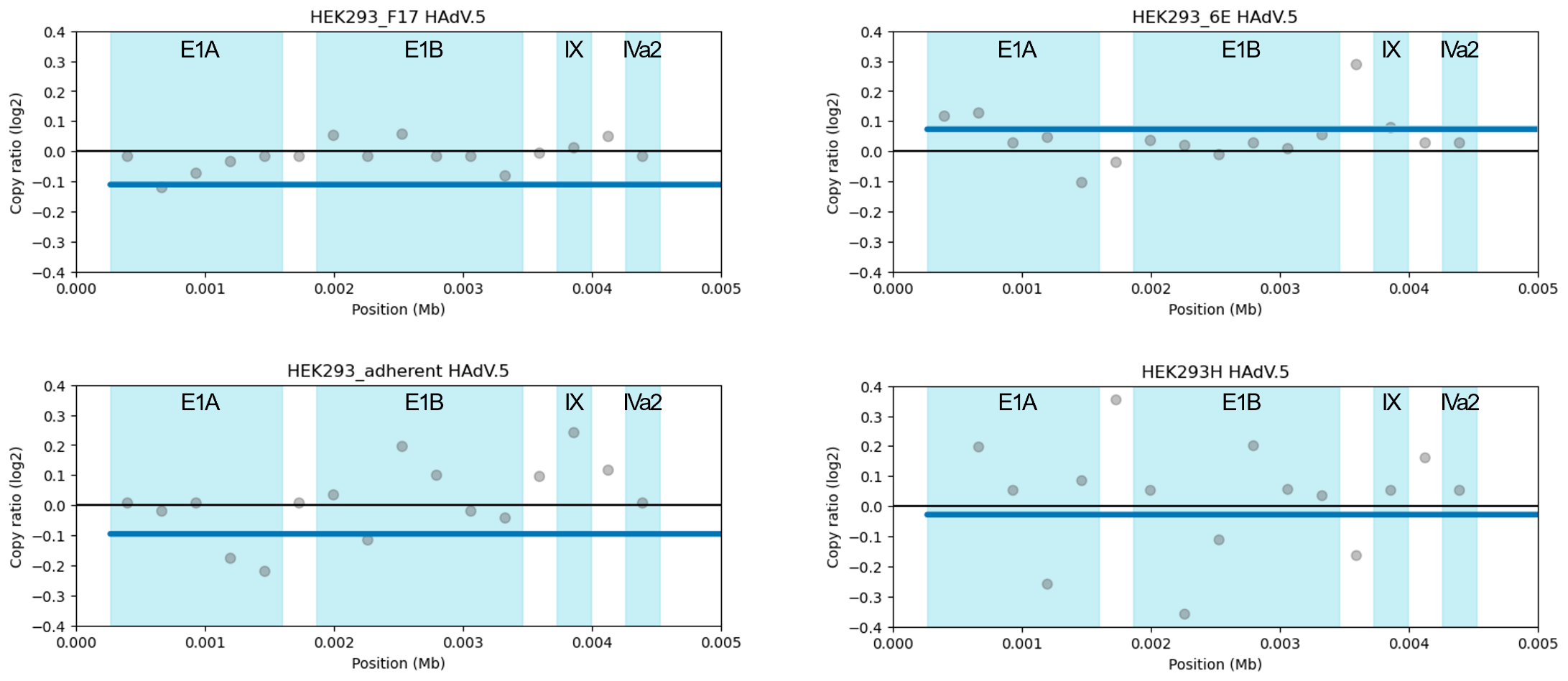


**Supplementary Figure 13**: Copy number variation analysis of integrated adenoviral region for suspension adapted cell line HEK293_F17, truncated EBNA1-expressing suspension cell line HEK293_6E, internal parental, adherent cell line HEK293_adherent, and clonally selected suspension cell line HEK293H. The scatter plot reveals calculated bins across the integrated segment of the human adenovirus 5 genome (HAdV.5). Values are presented on a log2 scale, with 0 corresponding to the diploid state, positive values indicate gains, and negative values indicate losses. Trend in copy number alteration is highlighted by a blue horizontal line. Light blue shaded areas reflect the open reading frames of the corresponding human adenoviral genes.


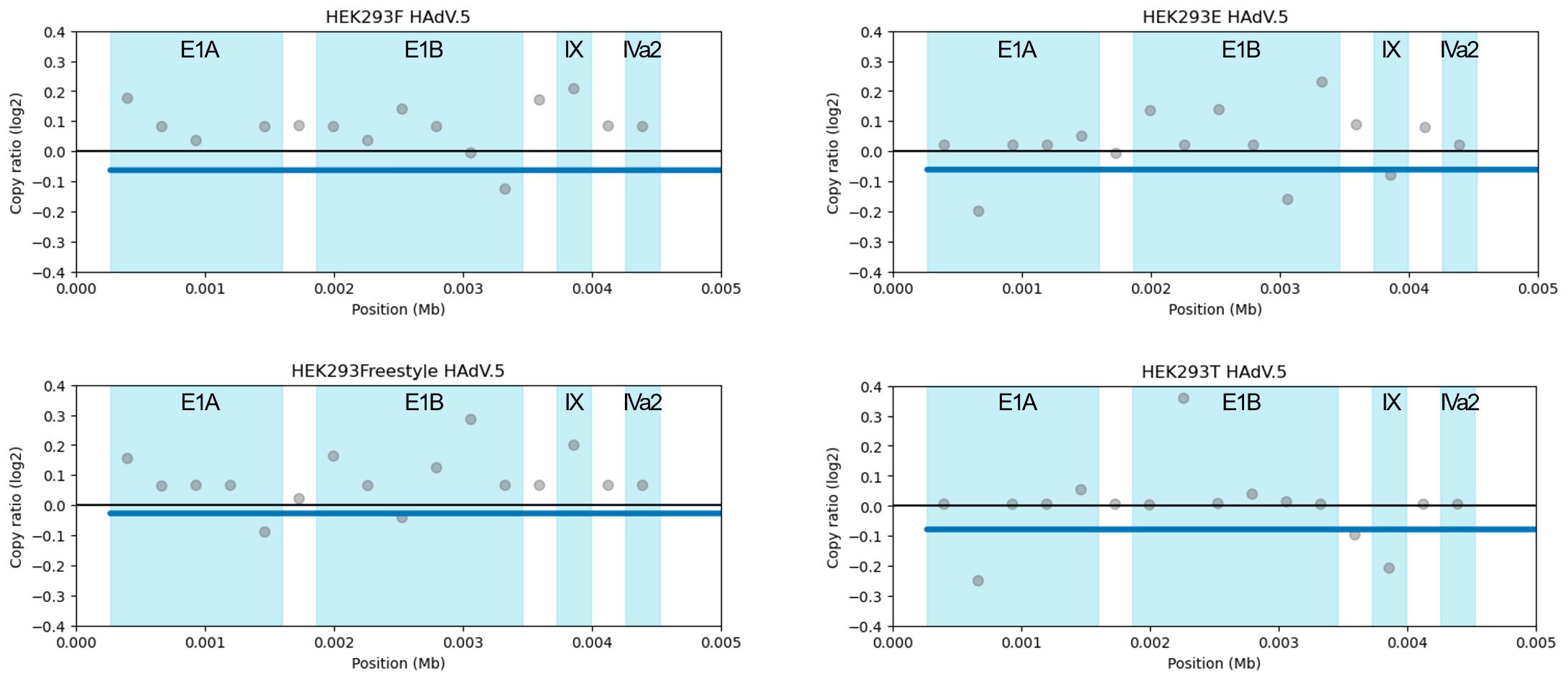


**Supplementary Figure 14**: Copy number variation analysis of integrated adenoviral region for clonally selected suspension cell line HEK293F, EBNA1-expressing adherent cell line HEK293E, medium adapted suspension cell line HEK293Freestyle and large T-antigen expressing adherent cell line HEK293T. The scatter plot reveals calculated bins across the integrated segment of the human adenovirus 5 genome (HAdV.5). Values are presented on a log2 scale, with 0 corresponding to the diploid state, positive values indicate gains, and negative values indicate losses. Trend in copy number alteration is highlighted by a blue horizontal line. Light blue shaded areas reflect the open reading frames of the corresponding human adenoviral genes.


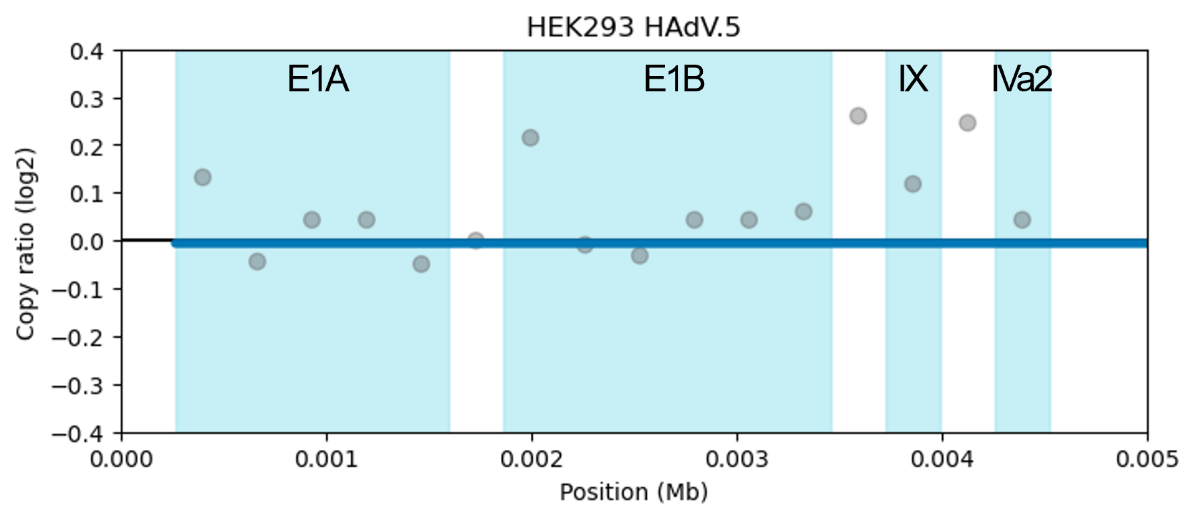


**Supplementary Figure 15**: Copy number variation analysis of integrated adenoviral region for external parental, adherent cell line HEK293. The scatter plot reveals calculated bins across the integrated segment of the human adenovirus 5 genome (HAdV.5). Values are presented on a log2 scale, with 0 corresponding to the diploid state, positive values indicate gains, and negative values indicate losses. Trend in copy number alteration is highlighted by a blue horizontal line. Light blue shaded areas reflect the open reading frames of the corresponding human adenoviral genes.

## Detection of human adenovirus 5 integration site

Realignment of chimeric reads, containing both viral and human sequences, to the human reference genome precisely confirmed the integration site of human adenovirus 5, which had also been identified by the structural variant caller (Manta) and previously described in literature [2]. The associated 19 bp deletion at the integration site within the pregnancy-specific β-1-glycoprotein 4 (PSG4) gene was also accurately validated.

The following coverage plots display the realigned chimeric reads along the human reference sequence of chromosome 19 at the specified positions for all 13 observed samples. Differences in depth of coverage reflect variations in sequencing depth between the two compared datasets.


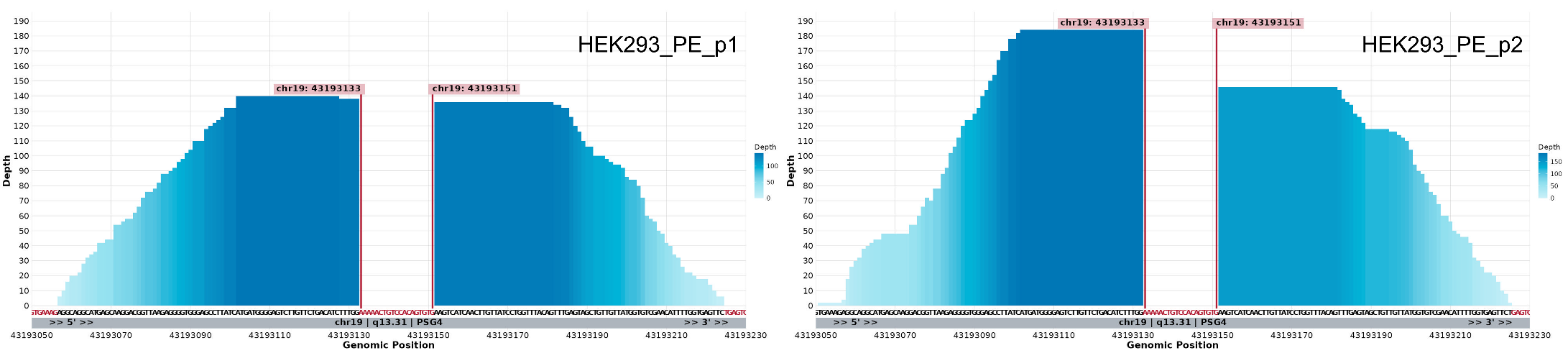


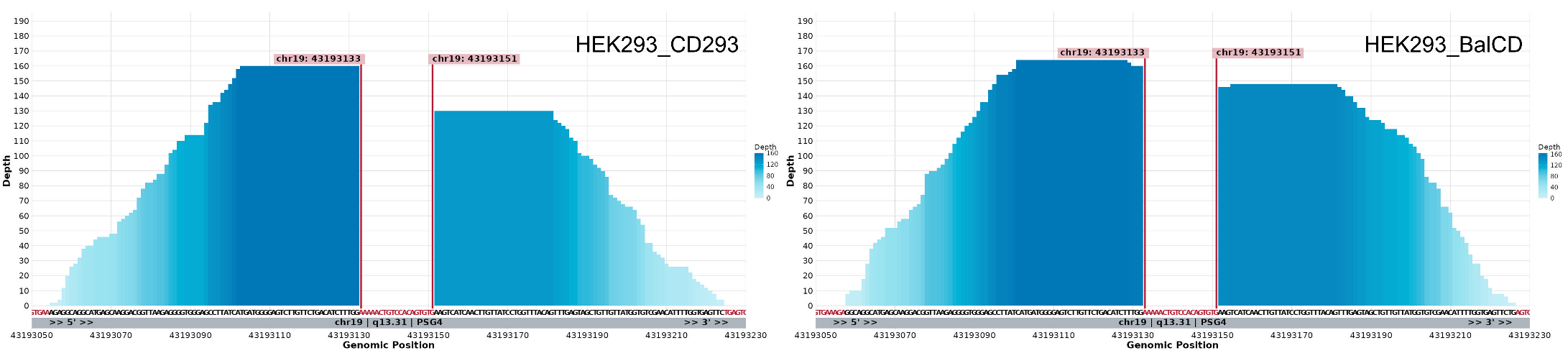


**Supplementary Figure 16**: Depth of covered regions at site of viral integration after realignment of chimeric reads. The red marked sequences indicate a 19 bp deletion at the site of integration, while the red flags denote the start and end points of integration on the human reference. The location is specified below, on the long arm of chromosome 19 at band 13.31, within the PSG4 gene.


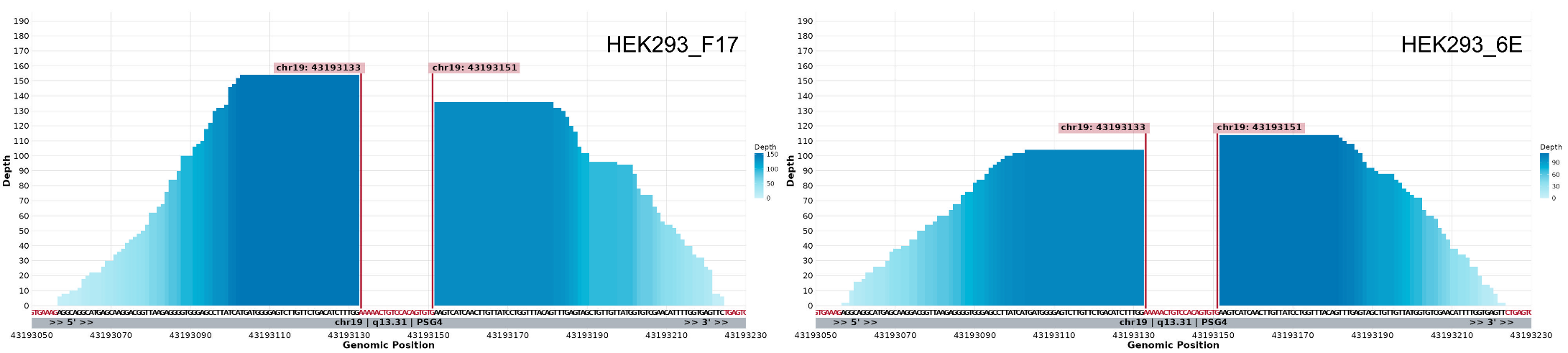


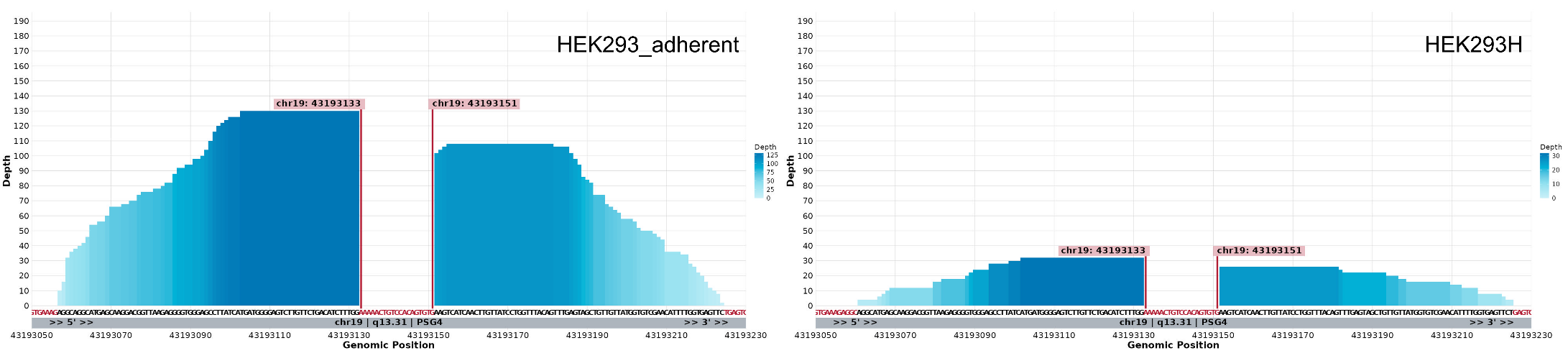


**Supplementary Figure 17**: Depth of covered regions at site of viral integration after realignment of chimeric reads. The red marked sequences indicate a 19 bp deletion at the site of integration, while the red flags denote the start and end points of integration on the human reference. The location is specified below, on the long arm of chromosome 19 at band 13.31, within the PSG4 gene.


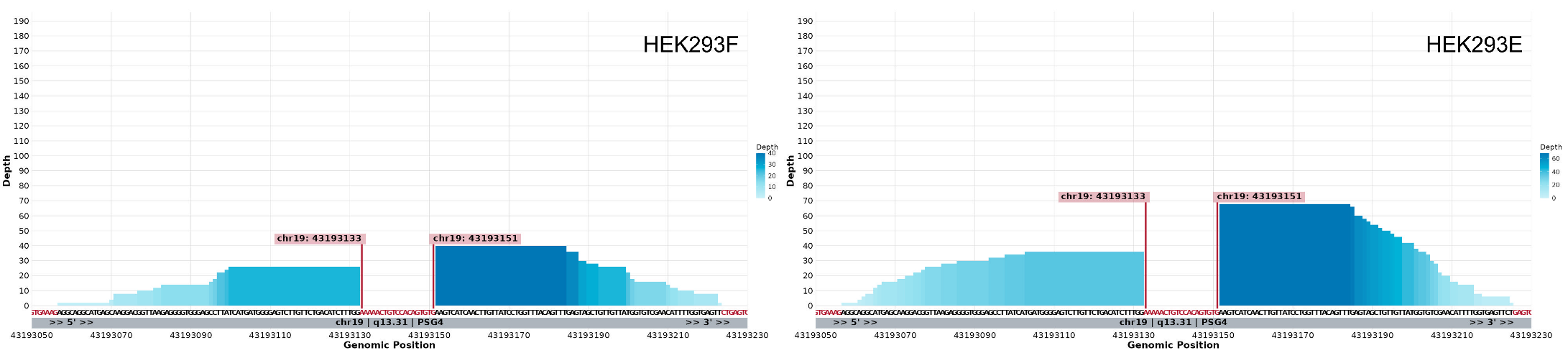


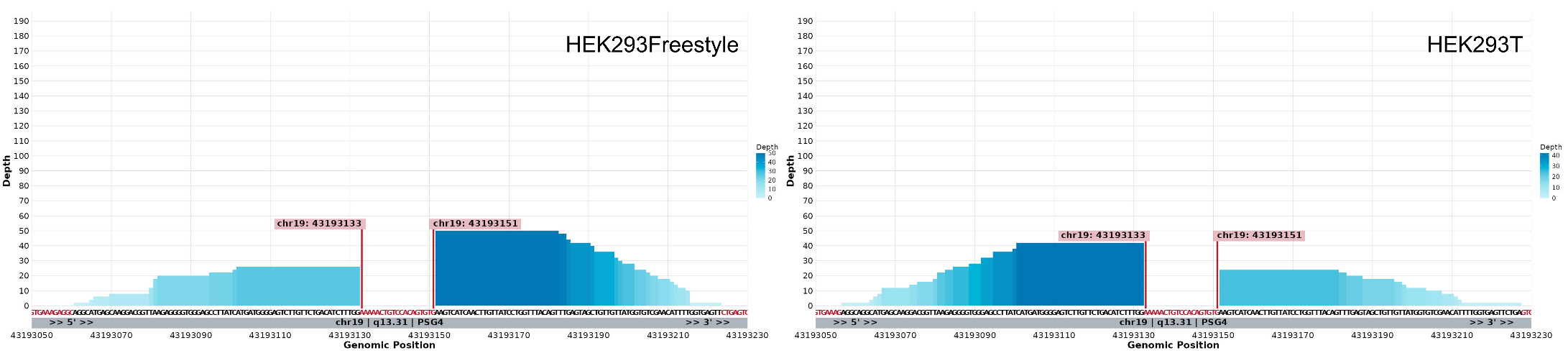


**Supplementary Figure 18**: Depth of covered regions at site of viral integration after realignment of chimeric reads. The red marked sequences indicate a 19 bp deletion at the site of integration, while the red flags denote the start and end points of integration on the human reference. The location is specified below, on the long arm of chromosome 19 at band 13.31, within the PSG4 gene.


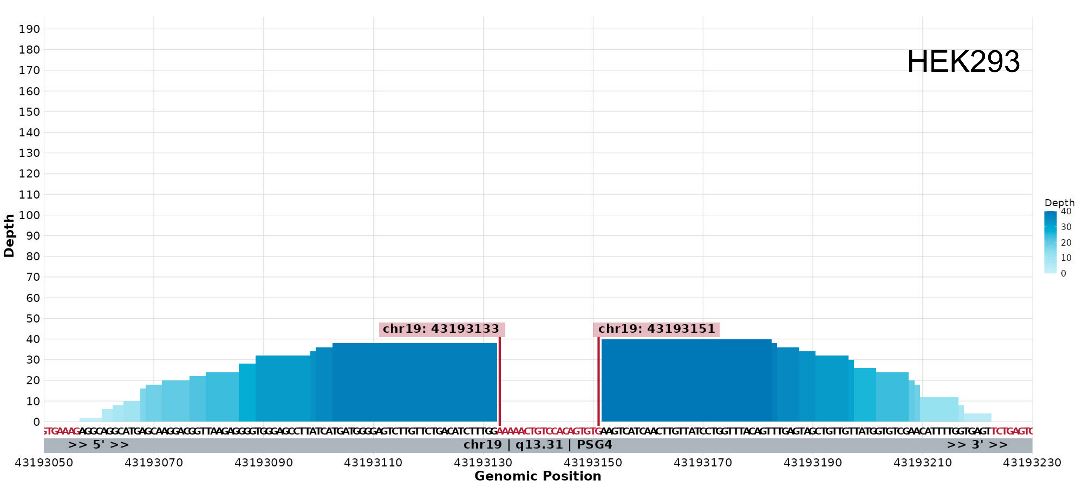


**Supplementary Figure 19**: Depth of covered regions at site of viral integration after realignment of chimeric reads. The red marked sequences indicate a 19 bp deletion at the site of integration, while the red flags denote the start and end points of integration on the human reference. The location is specified below, on the long arm of chromosome 19 at band 13.31, within the PSG4 gene.

## Additional information

Detailed information and numerical results related to the described data preparation steps and analyses are provided in Supplementary Tables 1 to 9.

**Supplementary Table 1:** SNP rates across the genomes

**Supplementary Table 2:** Indel rates across the genomes

**Supplementary Table 3:** GO term analysis results and settings for common core mutations

**Supplementary Table 4:** GO term analysis results and settings for common large structural rearrangements

**Supplementary Table 5:** GO term analysis results and settings for suspension associated variants (default)

**Supplementary Table 6:** Description of 45 enriched genes GO term results of suspension associated variants (default)

**Supplementary Table 7:** GO term analysis results and settings for suspension associated variants (reduced significance threshold)

**Supplementary Table 8:** Excerpt from alignment metrics report

**Supplementary Table 9:** Total and filtered SV counts
